# Nuclear versus mitochondrial DNA: evidence for hybridization in colobine monkeys

**DOI:** 10.1186/1471-2148-11-77

**Published:** 2011-03-24

**Authors:** Christian Roos, Dietmar Zinner, Laura S Kubatko, Christiane Schwarz, Mouyu Yang, Dirk Meyer, Stephen D Nash, Jinchuan Xing, Mark A Batzer, Markus Brameier, Fabian H Leendertz, Thomas Ziegler, Dyah Perwitasari-Farajallah, Tilo Nadler, Lutz Walter, Martin Osterholz

**Affiliations:** 1 Primate Genetics Laboratory, German Primate Center, Kellnerweg 4, 37077 Göttingen, Germany; 2 Gene Bank of Primates, German Primate Center, Kellnerweg 4, 37077 Göttingen, Germany; 3 Cognitive Ethology Laboratory, German Primate Center, Kellnerweg 4, 37077 Göttingen, Germany; 4 Departments of Statistics and Evolution, Ecology and Organismal Biology, The Ohio State University, Columbus, Ohio 43210, USA; 5 Reproductive Biology Unit, German Primate Center, Kellnerweg 4, 37077 Göttingen, Germany; 6 Department of Anatomical Sciences, State University of New York, Stony Brook, New York 11794-8081, USA; 7 Department of Human Genetics, University of Utah, 15 North 2030 East, Salt Lake City, Utah 84112, USA; 8 Department of Biological Sciences, Louisiana State University, 202 Life Sciences Building, Baton Rouge, Louisiana 70803, USA; 9 Research Group Emerging Zoonoses, Robert Koch Institute, Postfach 650261, 13302 Berlin, Germany; 10 Primate Research Center and Department of Biology, Bogor Agricultural University, Jl. Lodaya II/5, Bogor 16151, Indonesia; 11 Frankfurt Zoological Society, Endangered Primate Rescue Center, Cuc Phuong National Park, Nho Quan District, Ninh Binh Province, Vietnam; 12 Stem Cell Biology Unit, German Primate Center, Kellnerweg 4, 37077 Göttingen, Germany

## Abstract

**Background:**

Colobine monkeys constitute a diverse group of primates with major radiations in Africa and Asia. However, phylogenetic relationships among genera are under debate, and recent molecular studies with incomplete taxon-sampling revealed discordant gene trees. To solve the evolutionary history of colobine genera and to determine causes for possible gene tree incongruences, we combined presence/absence analysis of mobile elements with autosomal, X chromosomal, Y chromosomal and mitochondrial sequence data from all recognized colobine genera.

**Results:**

Gene tree topologies and divergence age estimates derived from different markers were similar, but differed in placing *Piliocolobus/Procolobus *and langur genera among colobines. Although insufficient data, homoplasy and incomplete lineage sorting might all have contributed to the discordance among gene trees, hybridization is favored as the main cause of the observed discordance. We propose that African colobines are paraphyletic, but might later have experienced female introgression from *Piliocolobus*/*Procolobus *into *Colobus*. In the late Miocene, colobines invaded Eurasia and diversified into several lineages. Among Asian colobines, *Semnopithecus *diverged first, indicating langur paraphyly. However, unidirectional gene flow from *Semnopithecus *into *Trachypithecus *via male introgression followed by nuclear swamping might have occurred until the earliest Pleistocene.

**Conclusions:**

Overall, our study provides the most comprehensive view on colobine evolution to date and emphasizes that analyses of various molecular markers, such as mobile elements and sequence data from multiple loci, are crucial to better understand evolutionary relationships and to trace hybridization events. Our results also suggest that sex-specific dispersal patterns, promoted by a respective social organization of the species involved, can result in different hybridization scenarios.

## Background

With more than 50 species and due to some ecological adaptations, such as a ruminant-like chambered stomach to digest food rich in fiber, the Old World monkey subfamily Colobinae represents a diverse and enigmatic group of primates [1,2]. Colobines are predominantly arboreal and occur in forest and woodland habitats. They have experienced two major radiations, one in Africa with the genera *Procolobus*, *Piliocolobus *and *Colobus*, and a second in South and Southeast Asia comprising the langur genera *Semnopithecus*, *Trachypithecus *and *Presbytis*, and the odd-nosed monkey genera *Rhinopithecus*, *Pygathrix*, *Nasalis *and *Simias *[2]. However, their phylogenetic relationships are disputed [3-7], and recent molecular studies detected substantial gene tree discordance [8-10].

Traditionally, African and Asian genera are believed to form reciprocally monophyletic groups [1,2,11,12], though paraphyly has also been proposed [3-5]. Molecular investigations clearly confirm a common origin of Asian colobines and the odd-nosed monkey group [8-10], but evidence for monophyly of the langur group as well as for African colobines is still lacking. Moreover, nuclear and mitochondrial data indicate conflicting relationships among langur genera, and between langurs and the odd-nosed monkeys [8-10]. While nuclear data consistently link *Semnopithecus *and *Trachypithecus *to the exclusion of all other Asian colobines [9,10], mitochondrial data either do not resolve these relationships [9] or suggest a clade consisting of *Presbytis *and *Trachypithecus *[8].

Incongruent phylogenetic relationships among genes, like those detected among colobines are common in phylogenetic studies and could be explained by homoplasy, insufficient data, nucleotide composition, differential lineage sorting, or hybridization [13-21]. To ascertain which of these possibilities are responsible for the incongruence, information from various independent molecular loci can be helpful [22]. To date, only mitochondrial and X chromosomal data as well as presence/absence information of mobile elements, all based on an incomplete taxon sampling, are available for comparative phylogenetic studies in colobines [8-10,23]. Among all marker systems, mobile element insertions are a promising tool to uncover phylogenetic relationships among colobine genera. Compared to sole sequence data, mobile elements such as Short Interspersed Elements (SINEs) and Long Interspersed Elements (LINEs) exhibit advantages which make them ideal markers for phylogenetic reconstructions (for review see [24-30]). Accordingly, mobile elements are successfully applied in numerous primate phylogenetic studies [9,28,31-39].

In our study, we examined the presence/absence pattern of mobile elements and compared the inferred phylogeny with those derived from mitochondrial and nuclear sequence data (in total ~30,000 bp per genus). We extended available X chromosomal and mitochondrial genome data, and sequenced de novo five autosomal loci that map to different human chromosomes, and six Y chromosomal loci from all ten colobine genera. By combining results from different marker systems, we provide detailed insights into the evolutionary and biogeographic history of colobine monkeys, and show that different hybridization mechanisms might have been involved during the colobine radiation.

## Results

### Nuclear phylogeny

**Figure 1:**
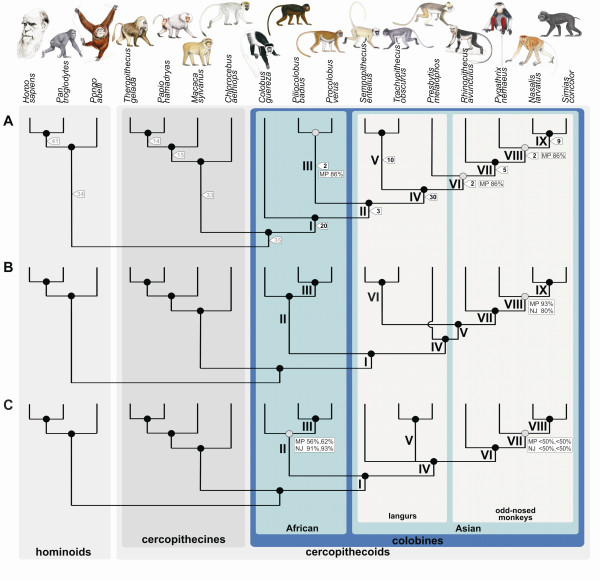
**Phylogenetic relationships among colobine and outgroup genera as inferred from different datasets**. Panels refer to insertions of mobile elements (A), combined nuclear sequence data (B), and mitochondrial genome data (C). Roman numbers are used as branch identifiers and are discussed in the text. In A, numbers in flags represent the number of available mobile elements (black: colobine markers, grey: non-colobine markers). In B and C, all nodes are significantly supported by ML and Bayesian reconstructions (≥95%, 1.0). Black and grey dots on nodes indicate high (≥95%) and lower (<95%) branch support as obtained from MP (in A-C) and NJ (in B and C) reconstructions, respectively. Bootstrap values <95% are presented at respective nodes. In C, first and second values refer to those obtained from reconstructions using datasets mtDNA1 and mtDNA2, respectively.

Eighty-three mobile elements are phylogenetically informative for colobines (Figure 1A, Additional file 1). Each of the following clades is strongly supported by at least five integrations: all colobines (clade I [A-I]), Asian colobines (A-IV), odd-nosed monkeys (A-VII), *Trachypithecus *and *Semnopithecus *(A-V), and *Nasalis *and *Simias *(A-IX). Three integrations were found in *Piliocolobus *and *Procolobus *and all Asian colobines (A-II), but not in *Colobus*. Two insertions suggested a sister grouping of *Procolobus *and *Piliocolobus *(A-III), *Presbytis *and the odd-nosed monkeys (A-VI), and a basal position of *Rhinopithecus *among the latter (A-VIII). Based on maximum-parsimony (MP) bootstrap analysis, most relationships were strongly supported (≥95%). Only the *Piliocolobus*/*Procolobus *(A-III), *Presbytis*/odd-nosed monkey (A-VI), and *Pygathrix*/*Nasalis*/*Simias *(A-VIII) clades gained relatively weak bootstrap values (86%). Based on alternative tree topology tests, different positions of the *Piliocolobus/Procolobus *clade and *Presbytis *among colobines were not rejected (*P *> 0.05), while relationships other than the most likely one were significantly rejected for all other taxa (*P *< 0.001, *P *< 0.05) (Additional file 2).

Next, we performed phylogenetic analyses based on the concatenated nuclear sequence dataset, including five autosomal loci, six Y chromosomal loci and a fragment of the X chromosomal Xq13.3 region (see Methods for detailed locus description). We combined all nuclear sequence data, because heuristic search methods for individual loci produced no conflicting relationships (Additional file 3), and partition homogeneity tests revealed no significant difference in their evolutionary history (Y chromosomal loci combined: *P *= 0.2939; autosomal loci combined: *P *= 0.1543; all nuclear loci combined: *P *= 0.3559). Nucleotide composition of studied species was similar (Additional file 4). Phylogenetic reconstructions yielded identical and significantly supported branching patterns irrespectively of the applied algorithm (MP, neighbor-joining [NJ], maximum-likelihood [ML], Bayesian) (Figure 1B, for a phylogram see Additional file 5). Only the *Pygathrix*/*Nasalis*/*Simias *(B-VIII) clade had lower support values (MP: 93%, NJ: 80%, but ML: 98%, Bayesian posterior probabilities [PP]: 1.0). The resultant tree topology was mainly congruent with the mobile element-based phylogeny, but two cases of incongruence were obvious. First, in the nuclear sequence-based phylogeny, African (B-II) and Asian (B-IV) colobine genera formed reciprocally monophyletic clades and second, *Presbytis *represented a sister lineage to the other Asian genera (B-V). According to alternative tree topology tests (Additional file 2), paraphyly of African colobines with *Piliocolobus*/*Procolobus *being closer related to Asian colobines than to *Colobus *as well as various alternative positions of *Presbytis *among Asian colobines were not rejected (*P *> 0.05). However, affiliations of *Presbytis *to either *Semnopithecus *or *Trachypithecus *were rejected (*P *< 0.001).

Estimated divergence ages from the combined nuclear dataset (Table 1) and single loci (Additional file 6), both based on an a-priori fixed tree topology as obtained from mobile elements, differed slightly, most likely due to the general low variability in the studied loci (Additional file 7). However, estimates were in the same range suggesting that loci evolve at similar evolutionary rates (Additional file 8). According to our nuclear estimates, *Colobus *and *Piliocolobus/Procolobus *successively split off from Asian genera 10.93 million years ago (mya) and 10.73 mya, respectively (for 95% highest posterior densities see Table 1). The latter two separated 6.92 mya. In Asia, an initial split occurred 8.12 mya and led to a clade consisting of *Trachypithecus *and *Semnopithecus*, and a group containing *Presbytis *and the odd-nosed monkeys. Among the latter, *Presbytis *diverged 7.96 mya and the odd-nosed monkeys began differentiating 6.43 mya. The most recent splits among Asian genera occurred between *Trachypithecus *and *Semnopithecus *(2.56 mya) and between *Nasalis *and *Simias *(1.06 mya).

**Table 1:** Estimation of divergence ages in mya (95% highest posterior density)

node	nuclear DNA	mitochondrial DNA
cercopithecoids - hominoids	24.39 (22.44-26.47)	23.73 (21.88-25.94)
*Pongo - Homo/Pan*	13.89 (12.80-14.95)	13.58 (12.51-14.64)
*Homo *- *Pan*	6.39 (5.85-7.01)	6.18 (5.62-6.70)
cercopithecines - colobines	15.50 (14.45-16.56)	15.92 (14.11-17.79)
*Cholorocebus - *other cercopithecines	9.47 (7.52-11.57)	10.56 (8.78-12.29)
*Macaca *- *Papio/Theropithecus*	6.59 (5.12-8.27)	8.55 (6.82-10.03)
*Papio - Theropithecus*	3.80 (3.20-4.38)	3.97 (3.39-4.46)
*Colobus *- other colobines (A-I)	10.93 (9.60-12.31)	-
*Piliocolobus/Procolobus *- Asian colobines (A-II)	10.73 (9.38-12.04)	-
African - Asian colobines (C-I)	-	10.90 (9.34-12.44)
*Colobus *- *Piliocolobus/Procolobus *(C-II)	-	8.47 (6.83-9.88)
*Piliocolobus - Procolobus *(A-III, C-III)	6.92 (4.38-9.35)	6.58 (4.99-8.04)
Asian colobines (A-IV, C-IV)	8.12 (7.14-9.16)	8.91 (7.43-10.23)
*Trachypithecus *- *Semnopithecus *(A-V)	2.56 (1.25-4.22)	-
*Presbytis *- odd-nosed monkeys (A-VI)	7.96 (6.93-8.95)	-
*Presbytis *- *Trachypithecus *(C-V)	-	7.45 (5.88-8.86)
odd-nosed monkeys (A-VII, C-VI)	6.43 (5.03-7.75)	6.91 (5.60-8.20)
*Pygathrix - Nasalis/Simias *(A-VIII, C-VII)	5.66 (4.22-7.01)	6.23 (5.11-7.38)
*Nasalis *- *Simias *(A-IX, C-VIII)	1.06 (0.44-1.81)	1.88 (1.21-2.45)

### Mitochondrial phylogeny

Mitochondrial and nuclear datasets were not combined, because the partition homogeneity test suggested that both track different evolutionary histories (*P *= 0.0002). Thus, mitochondrial sequence data were analyzed separately. For both alignments (mtDNA1, mtDNA2; for details about alignments see Methods), we observed a major shift in nucleotide composition between colobine and non-colobine representatives (Additional file 4). Both alignments produced identical and significantly supported branching patterns among genera (Figure 1C, for a phylogram see Additional file 5). Only the *Pygathrix*/*Nasalis*/*Simias *(C-VII) and African colobine (C-II) clades gained low MP (<50%, <50%, 56%, 62%) and NJ (<50%, <50%, 91%, 93%) bootstrap values, but ML and Bayesian reconstructions provided strong support for both nodes (96%, 100%; 1.0, 1.0). In principal, the tree topology was identical to those obtained from mobile elements and nuclear sequence data. However, as in the nuclear sequence tree, mitochondrial data suggested African (C-II) and Asian (C-IV) colobines as reciprocal monophyletic clades. Moreover, Asian colobines further diverged into a lineage leading to the odd-nosed monkeys (C-VI), a lineage comprising *Trachypithecus *and *Presbytis *(C-V), and finally a lineage with solely *Semnopithecus*, while the relationships among these three lineages remained unresolved.

According to alternative tree topology tests, paraphyly of African colobines with *Piliocolobus*/*Procolobus *being closer related to Asian colobines than to *Colobus *was rejected (*P *< 0.001, Additional file 2). Among Asian colobines, relationships in which *Trachypithecus *and *Presbytis *do not form a monophyletic clade were also rejected (*P *< 0.001, *P *< 0.05), as well as a close relationship of *Trachypithecus *and *Semnopithecus *(*P *< 0.01). In contrast, different positions of *Semnopithecus *among Asian colobines were similarly likely (*P *> 0.05).

Divergence age estimates from mitochondrial data were similar to nuclear estimates in case where identical branching patterns were obtained (Table 1). According to mitochondrial data, African and Asian colobine lineages were separated 10.90 mya. In Africa, *Colobus *represents the first split (8.47 mya), followed by the divergence of *Piliocolobus *and *Procolobus *(6.58 mya). The major Asian split leading to the three lineages *Semnopithecus*, *Trachypithecus*/*Presbytis *and the odd-nosed monkeys occurred 8.91 mya. *Trachypithecus *diverged from *Presbytis *7.45 mya. The diversification of odd-nosed monkeys into genera started 6.91 mya and ended with the split between *Nasalis *and *Simias *1.88 mya.

### Inferring hybridization in the presence of incomplete lineage sorting

To assess the possible reasons for the incongruence between the nuclear and mitochondrial trees, we applied the method proposed by Kubatko [40]. The method assumes that incomplete lineage sorting (ILS) explains observed gene tree incongruence to some extent, and seeks to determine whether all variation in observed gene trees can be explained by ILS alone, as modeled by the coalescent process, or whether hybridization helps to explain significantly more the observed variation. Then, the Akaike information criterions (AIC) in each model (may or may not include hybridization scenarios) were compared to determine the best-fit model. For our data, two possible hybridization events were hypothesized. The first involved *Trachypithecus*, with parental taxa *Semnopithecus *and *Presbytis*, while the second involved the clade containing *Piliocolobus *and *Procolobus*, *Colobus *and the ancestor of Asian colobines.

**Figure 2:**
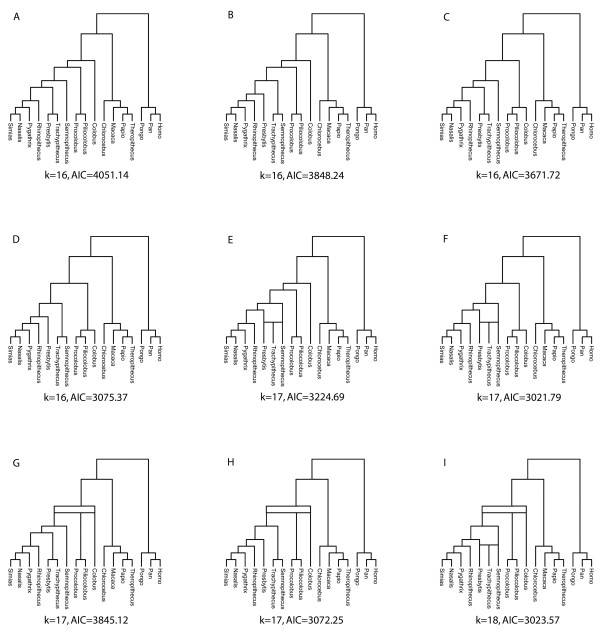
**The nine alternative hybridization scenarios compared in the coalescent framework**. Beneath each tree, the number of parameters in the model (k) is given as well as the AIC. The lowest AIC values are observed for trees F and I, which indicate a similar fit for these scenarios.

By comparing the results from models with or without the hybridization events, the best-fit model (AIC = 3021.79, Figure 2F) was a tree in which *Trachypithecus *is the result of hybridization between *Presbytis *and *Semnopithecus*. The second best-fit model (AIC = 3023.57, Figure 2I) comprised the tree that includes both tested hybridization events. AIC values for all seven other models were considerably higher (3072.25 - 4051.14). Since AIC values for the scenarios presented in Figures 2F and 2I were the lowest and were within 2 of one another, both were considered plausible explanations for the observed gene tree discordances [41]. It is worth pointing out that the model used here to compute the AIC assumes that ILS is a possible source of gene trees incongruence. Since the two best-fit models include at least one hybridization event, it is clear that ILS alone does not adequately describe the extent of incongruence in the observed gene trees.

## Discussion

By combining presence/absence analysis of mobile elements with autosomal, X chromosomal, Y chromosomal and mitochondrial sequence data, the present study provides comprehensive insights into the evolutionary history of colobines. Most relationships are resolved and strongly supported by mobile elements and sequence data. Moreover, relationships and estimated divergence ages as obtained from different datasets are mainly congruent and in agreement with earlier studies [8-10,23,42-45]. Our study, however, also reveals significant discrepancies among gene trees. First, mitochondrial and nuclear sequence data suggest a monophyletic African colobine clade, while mobile elements provide evidence for a closer connection of the *Piliocolobus*/*Procolobus *clade to Asian genera than to *Colobus*. Second, mobile elements indicate close relationships between *Semnopithecus *and *Trachypithecus*, and between *Presbytis *and the odd-nosed monkeys. Nuclear sequence data support the former clade, but suggest *Presbytis *as basal among Asian colobines. In contrast, in the mitochondrial phylogeny, *Presbytis *and *Trachypithecus *are displayed as sister lineages, while the position of *Semnopithecus *remains ambiguous.

### Possible explanations for gene tree discordance

Inadequate data, homoplasy, nucleotide composition, ILS or hybridization could be potential explanations for the observed differences [13-21]. For the mitochondrial dataset, at least for the African and *Presbytis*/*Trachypithecus *clades, incorrect branching patterns due to inadequate data or homoplasy are unlikely, since sufficient phylogenetic resolution with long internal branches is obtained. Likewise, a shift in nucleotide composition and differential sorting of ancestral mitochondrial lineages is implausible. Since the major shift in nucleotide composition was detected between colobines and non-colobines, it cannot be responsible for gene tree discordances among colobines. If the African and *Presbytis*/*Trachypithecus *clades are indeed the result of incomplete sorting of mitochondrial lineages, the mitochondrial divergence between respective genera should predate the nuclear splitting times, which is not the case (African colobines: 10.93 mya nuclear vs. 8.47 mya mitochondrial; *Presbytis - Trachypithecus*: 8.12 mya nuclear vs. 7.45 mya mitochondrial). However, the unresolved position of *Semnopithecus *among Asian colobines might have been affected by one or several of the above mentioned factors, or alternatively, might be the result of a true radiation-like divergence of lineages. For nuclear data, these factors are unlikely explanations as well for the branching of *Trachypithecus *and *Semnopithecus*, because ten independent insertions and sequence data from 12 nuclear loci clearly confirm their close relationship. More challenging are explanations for the discordant positions of *Presbytis *and the African genera among colobines in phylogenies revealed by mobile elements and nuclear sequence data. Mixed genomes due to differentially selected genes cannot be excluded, but interestingly, both mobile elements and nuclear sequence data (as revealed from single locus analysis) show no conflicting phylogenies themselves. Most prominent, however, the mobile element-based phylogeny is not rejected by nuclear sequence data, indicating that insufficient informative sites, as also suggested by the low resolution of phylogenetic relationships in single-locus analysis, in the latter dataset might display incorrect relationships. For the integration of mobile elements, homoplasy is typically regarded as minimal [25,28,30], but ILS has been reported [36,39]. Only two and three integrations support the branching of *Presbytis *with odd-nosed monkeys and the paraphyly of African colobines, and alternative relationships cannot be rejected statistically. However, no inconsistent elements were detected and subtractive hybridizations specifically set up to screen for African colobine and *Trachypithecus*/*Presbytis *monophyly markers revealed no equivalent insertions. Accordingly, ILS seems to be an unreasonable explanation for our findings. Since the mobile element-based phylogeny is not rejected by nuclear sequence data and due to their reliability as molecular-cladistic markers, the phylogeny suggested by mobile elements is assumed to reflect the true nuclear phylogeny of colobines, although we explicitly note that mosaic genomes cannot be excluded.

Because all above-mentioned factors provide no sufficient explanation for the herein detected discordances between mitochondrial and nuclear phylogenies, we favor ancestral hybridization as the main reason for the discordant pattern. Furthermore, comparisons of models with and without hybridization in a model selection framework strongly support hybridization in the presence of ILS over models of ILS alone. In other words, even after ILS was taken into account as a factor in the observed incongruence among gene trees, we still found support for hybridization in the evolutionary history of these taxa. This refers at least to Asian colobines, but hybridization among African colobines cannot be excluded either by the method we applied here.

### Hybridization hypothesis

Although bidirectional hybridization, which would be indicated by mixed genomes, cannot be excluded with our data, a female introgression event is hypothesized for African colobines. The direction of gene flow remains obscure due to the rapid diversification of the colobine ancestor in Africa, but female introgression from *Piliocolobus/Procolobus *into *Colobus *is indicated and gains further support by some biological data [1,2]. In contrast to *Colobus*, females in *Piliocolobus *and *Procolobus *tend to leave their natal groups, which was most likely also the case in their ancestor [1], and *Colobus *males are on average larger than *Piliocolobus *and *Procolobus *males [1], thus increasing the chance of hybridization between *Colobus *males and *Piliocolobus/Procolobus *females. Moreover, hybridization between both ancestral lineages is in principal possible, because (at least nowadays) they occur in sympatry over wide ranges of their distribution [1,2]. Accordingly, after the successive separation of *Colobus *and *Piliocolobus/Procolobus *from the Asian colobine ancestor, *Piliocolobus/Procolobus *females might have entered *Colobus *populations and hybridized with their males. Backcrossing of hybrid females with resident *Colobus *males might has led to the fixation of the *Piliocolobus/Procolobus *mitochondrial lineage in the hybrid population, while the original nuclear genome of *Colobus *increased again in every generation.

For Asian langurs, we propose male introgression from *Semnopithecus *into *Trachypithecus *followed by nuclear swamping. Both genera are similar in their morphology and general appearance [2,46,47], but males in *Semnopithecus *are larger than in *Trachypithecus *[1]. Moreover, hybridization events due to (at least nowadays) partially overlapping ranges are generally possible [1,2]. Accordingly, after an initial separation, *Semnopithecus *males, which leave their natal group like most other primate males [1,48], might have invaded *Trachypithecus *populations and hybridized successfully with the resident females. By backcrossing with further invading *Semnopithecus *males over a longer period, the *Trachypithecus *population might have accumulated nuclear material of *Semnopithecus *(nuclear swamping), while the mitochondrial genome remained *Trachypithecus*-like.

### Biogeographic implications

**Figure 3:**
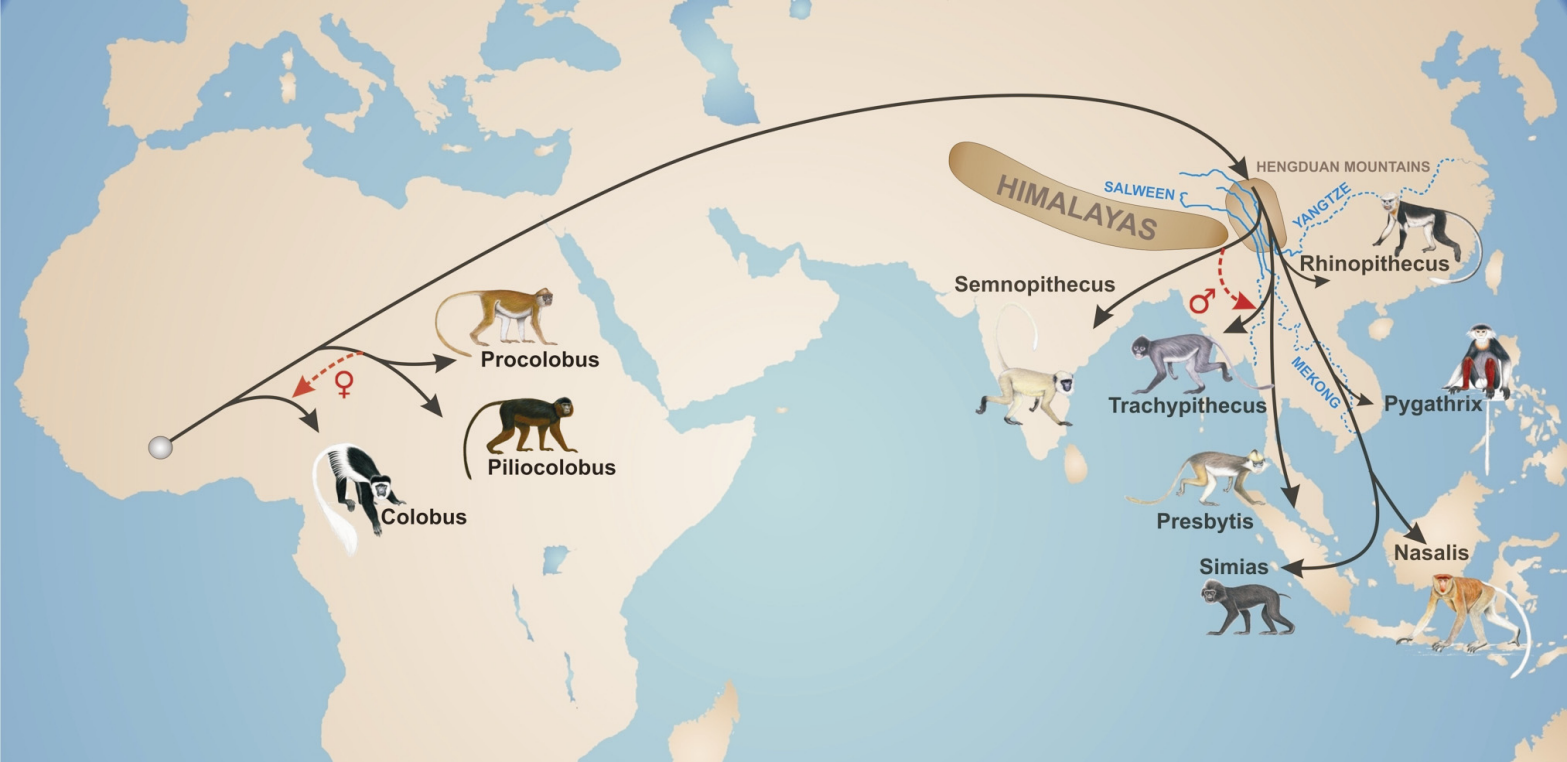
**Dispersal scenario for colobine monkeys**. Colobines most likely originated in western Africa. After the successive split of *Colobus *(~10.9 mya) and a progenitor of *Piliocolobus/Procolobus *(~10.7 mya) from the ancestor of Asian colobines, gene flow between both African lineages via female introgression from the *Piliocolobus/Procolobus *progenitor into *Colobus *occurred until ~8.5 mya (displayed by red-dashed arrow). During the late Miocene, colobines invaded eastern Asia most likely via a route north of the Himalayas. After their arrival at the Hengduan Mountains, Asian colobines diversified into a lineage comprising a progenitor of the odd-nosed monkeys and *Trachypithecus/Presbytis*, and of *Semnopithecus*, which later colonized the Indian subcontinent. Shortly afterwards, *Trachypithecus/Presbytis *split off from odd-nosed monkeys, and migrated to southern mainland Asia, before finally both genera diverged from each other. In the region of today's Burma, Bangladesh and India, *Semnopithecus *and *Trachypithecus *came into secondary contact and hybridized until ~2.6 mya (displayed by red-dashed arrow). In the latest Miocene, odd-nosed monkeys migrated from China to the south and expanded their range into Indochina and Sundaland. *Nasalis *and *Simias *finally separated from each other 1.1-1.9 mya.

By combining the available information, we develop the following extended dispersal scenario for colobines (Figure 3). The origin of the subfamily is most likely in Africa, which is in agreement with earlier suggestions [1,49]. On the African continent, *Colobus *split off first from the main stem ~10.93 mya, followed shortly afterwards by the progenitor of *Piliocolobus *and *Procolobus*. After this initial separation, hybridization between both lineages might have lasted until finally both mitochondrial lineages diverged (~8.47 mya). Presumably, respective splitting and hybridization events took place in western Africa, because all three genera occur there in sympatry [1,2], and the most ancient splits among *Piliocolobus *and *Colobus *species are also found there [45]. The Asian colobine ancestor most likely invaded Eurasia via an emerging land bridge connecting Africa and the Arabian Peninsula in the late Miocene [49,50]. Whether a route into eastern Asia north or south of the Himalayas was chosen is a matter of speculation, but north of the Himalayas, on the Tibetan plateau, colobine fossils from the late Miocene were found, which is not the case south of the Himalayas [1]. Although not confirmed, the Hengduan Mountains in the border region of today's Burma, India and China might have been a possible diversification hotspot [4,51,52]. In the region, all the larger Southeast Asian rivers (Mekong, Salween, Yangtze) rise, which are all well-known as barriers for arboreal primates [53] and are all known to exist since at least the early Miocene [54]. *Semnopithecus *might have diverged as first lineage and invaded the Indian subcontinent. Subsequently, the progenitor of *Presbytis *and *Trachypithecus *separated from the odd-nosed monkey ancestor and migrated into southern mainland Asia. Afterwards, *Presbytis *diverged from *Trachypithecus *and entered first the Malaysian peninsular and later on Sundaland during periods of lowered sea levels [55]. *Trachypithecus *and *Semnopithecus *came into secondary contact and might have hybridized until the earliest Pleistocene. A potential contact zone could be the region of today's Bangladesh, Burma and the northeast of India, which is suggested as hybridization area for several primate species [9,44,56]. On the Asian mainland, odd-nosed monkeys successively migrated from China to the south and expanded their range into Indochina and Sundaland in the latest Miocene. The migration into Sundaland was probably via land bridges connecting the mainland with Sundaland islands during periods of lowered sea levels [55]. Finally, *Nasalis *on Borneo and *Simias *on the Mentawai islands west of Sumatra diverged in the Pleistocene. Due to the dating discrepancy (mitochondrial data: 1.88 mya, nuclear data: 1.06 mya), further gene flow between both genera after the initial separation cannot be excluded, especially considering that migration was repeatedly possible via land bridge connections during the Pleistocene [55].

## Conclusion

Our study gives new and most comprehensive insights into the evolutionary history of colobine monkeys, and suggests hybridization among ancestral lineages as the most likely cause for the observed phylogenetic incongruences. Only the combination of maternally, paternally and bi-parentally inherited markers as well as the combination of sequence data with presence/absence patterns of mobile elements proved to be an adequate and reliable phylogenetic approach, particularly in revealing hybridization events. However, data from additional nuclear loci and a broader taxonomic sampling is required to fully understand hybridization mechanisms in colobines.

Hybridization among taxa is traditionally recognized as a factor leading to limited diversification, reproductive isolation and lowered fitness [57,58], whereas our and earlier studies clearly indicate that hybridization played a prominent role in diversification and speciation of primates (for review see [59,60]). Hybridization events are genetically confirmed within all major primate lineages, mainly among species (e.g., [56,61-65]) but also between genera (e.g., [9,44,66]). Even for the human lineage, hybridization has been suggested as an important evolutionary mechanism [67-69].

Since male dispersal and female philopatry predominates in primates [48], male introgression, and if intensive backcrossing of hybrids with more invading males occurs, followed by nuclear swamping would be the most likely hybridization scenario. In fact, the hybridization among Asian langur genera is most likely the result of such an event. However, as proposed for African colobines, alternative mechanisms (e.g. female introgression) could also occur, promoted by a respective social organization, where female migration predominates.

## Methods

### Sample collection and DNA extraction

Blood, tissue or fecal samples from representatives of all ten colobine genera (*Colobus*, *Piliocolobus*, *Procolobus*, *Presbytis*, *Trachypithecus*, *Semnopithecus, Rhinopithecus*, *Pygathrix*, *Nasalis*, *Simias*) and several non-colobine taxa (*Macaca*, *Papio, Theropithecus, Chlorocebus, Pongo*, *Pan*) were obtained from specimens kept in zoos or breeding facilities, or collected in the field (Table 2). Sample collection was conducted according to relevant German and international guidelines, including countries where we collected samples. Fecal samples were collected in a non-invasive way without disturbing, threatening or harming the animals. Blood samples were taken by veterinarians for diagnostic reasons to check the health status of the respective individuals, and tissue samples were obtained only from deceased specimens. Total genomic DNA was extracted with the DNeasy Blood & Tissue or QIAamp DNA Stool Mini kits from Qiagen following standard procedures.

**Table 2:** Origin and sample type of studied species

species	origin	sample type
*Colobus guereza*	Cologne zoo, Germany	tissue
*Piliocolobus badius*	Taï National Park, Ivory Coast	tissue
*Procolobus verus*	Taï National Park, Ivory Coast	tissue
*Semnopithecus entellus*	Dresden zoo, Germany	blood
*Trachypithecus obscurus*	Wuppertal zoo, Germany	blood
*Presbytis melalophos*	Howletts Wild Animal Park, Great Britain	tissue
*Pygathrix nemaeus*	Cologne zoo, Germany	tissue
*Rhinopithecus avunculus*	Endangered Primate Rescue Center, Vietnam	tissue
*Nasalis larvatus*	Wilhelma Stuttgart, Germany	blood
*Simias concolor*	Siberut Conservation Programme, Indonesia	feces
*Macaca sylvanus*	Nuremberg zoo, Germany	blood
*Papio hamadryas*	Munich zoo, Germany	blood
*Theropithecus gelada*	Duisburg zoo, Germany	blood
*Chlorocebus aethiops*	Paul-Ehrlich-Institute, Germany	blood
*Pongo abelii*	Nuremberg zoo, Germany	blood
*Pan troglodytes*	Munich zoo, Germany	blood

### Analysis of mobile elements

Due to their high copy number (~one million) and relatively small size (~300 bp), the primate specific *Alu *elements were selected as molecular-cladistic markers. The presence or absence of mobile elements in different colobines at specific loci was tested via PCR using primers occupying the flanking region of the insertion site. Details on analyzed loci, primers and presence/absence pattern of mobile elements in studied species are listed in Additional file 1. For most loci, sequencing was neglected, but in relevant cases the insertion orthology was confirmed by sequencing, and direct repeats flanking the insertion as well as the original target site prior to transposition were traced.

In our study, we included published markers [9,23,35], which were further examined in previously untested genera, and newly detected integration loci (Additional file 1). Therefore, we performed subtractive hybridizations following described methods [9]. To avoid biased hybridization results, various species combinations were used as tracer and driver (hybridization 1: tracer *Nasalis/Pygathrix*, driver *Presbytis*; hybridization 2: tracer *Nasalis/Pygathrix*, driver *Semnopithecus*; hybridization 3: tracer *Trachypithecus/Presbytis*, driver *Pygathrix*; hybridization 4: tracer *Presbytis*, driver *Semnopithecus*; hybridization 5: tracer *Piliocolobus/Colobus*, driver *Pygathrix*). Besides *Alu *insertions, a LINE present in *Piliocolobus *and *Procolobus *in the studied Xq13.3 fragment was additionally applied as marker (Additional file 1).

Phylogenetic reconstructions using the MP algorithm were conducted in PAUP v4.0b10 [70]. Presence of an integration was coded as 1, its absence as 0, and missing data as '?'. Internal node support was obtained via a heuristic search with 10,000 bootstrap replications. To evaluate the reliability of the depicted relationships among colobines, various alternative tree topologies (Additional file 2) were assessed with the Kishino-Hasegawa test [71] with full optimization and 1,000 bootstrap replications in PAUP.

### Amplification and sequencing of nuclear loci

Inter-exonic intron and exonic sequences were generated for six single-copy genes of the Y chromosome, five autosomal loci, and a fragment of the X chromosomal Xq13.3 region. With exception of the *SRY *gene (sex-reversal, Y chromosome), all other Y chromosomal loci (*DBY5*: Dead Box, intron 5; *SMCY7*: SMC mouse homologue, intron 7; *SMCY11*: SMC mouse homologue, intron 11; *UTY18*: ubiquitous TPR motif, intron 18; *ZFYLI*: Zinc finger, last intron) have homologues on the X chromosome (X degenerate). As autosomal loci, we selected intron 11 of the von Willebrand Factor (*vWF11*), located on human chromosome 12, intron 3 of the serum albumin gene (*ALB3*, human chromosome 4), intron 3 of the interstitial retinol-binding protein (*IRBP3*, human chromosome 10), intron 1 of the transition protein 2 (*TNP2*, human chromosome 16) and intron 1 of the transthyretin gene (*TTR1*, human chromosome 18). *SRY, DBY5, SMCY7, SMCY11, UTY18, vWF11 *and a ~4,300 bp fragment of the Xq13.3 region were amplified using primers and PCR conditions as described [10,72-75] (Additional file 9). For the amplification of *ZFYLI, ALB3, IRBP3, TNP2 *and *TTR1*, new primers (Additional file 9) were designed on the basis of available primate sequences in GenBank. PCR conditions for the latter comprised a pre-denaturation step at 94°C for 2 min, followed by 40 cycles each with denaturation at 94°C for 1 min, annealing at varying temperatures (Additional file 9) for 1 min, and extension at 72°C for 2 min. At the end, a final extension step at 72°C for 5 min was added. The results of all PCR amplifications were checked on 1% agarose gels. PCR products were cleaned with the Qiagen PCR Purification kit and subsequently sequenced on an ABI 3130 × l sequencer using the BigDye Terminator Cycle Sequencing kit. Alignments and sequences are available in TreeBASE (http://purl.org/phylo/treebase/phylows/study/TB2:S11179) and GenBank, respectively (for GenBank accession numbers see Additional file 10).

### Amplification and sequencing of mitochondrial genomes

To reduce the likelihood of amplifying nuclear pseudogenes (numts), complete mitochondrial genomes from four colobine genera (*Rhinopithecus*, *Pygathrix*, *Nasalis*, *Procolobus*) were generated following an approach in which two overlapping ~10,000 bp long fragments were amplified via long-range PCR [8,43]. Due to degradation of DNA extracted from faeces, the mitochondrial genome of *Simias *was amplified via five overlapping fragments, each with a size of ~5,000 bp. All long-range PCRs were performed with the SuperTaq Plus polymerase from Ambion following protocols of the supplier and primers as described [8,43]. Long-range PCR amplicons were separated on 1% agarose gels, excised from the gel, purified with the Qiagen Gel Extraction kit and used as template for nested PCRs. PCR conditions for all nested PCR amplifications were identical and comprised a pre-denaturation step at 94°C for 2 min, followed by 30 cycles each with denaturation at 94°C for 1 min, annealing at 60°C for 1 min, and extension at 72°C for 1.5 min. At the end, a final extension step at 72°C for 5 min was added. Nested PCR products (900-1,200 bp in length) were cleaned with the Qiagen PCR Purification kit and sequenced on an ABI 3130 × l sequencer. Sequences were assembled with Geneious v4.6.1 [76]. No inconsistent positions in overlapping regions were detected and all protein-coding genes were correctly translated. Annotation of mitochondrial genomes was conducted with the online program DOGMA [77] and manually inspected. Alignment and sequences are available in TreeBASE (http://purl.org/phylo/treebase/phylows/study/TB2:S11179) and GenBank, respectively (for GenBank accession numbers see Additional file 10).

### Statistical analysis of sequence data

For phylogenetic reconstructions, all datasets comprised 17 sequences including each one representative of the ten colobine genera (*Colobus*, *Piliocolobus*, *Procolobus*, *Trachypithecus*, *Semnopithecus*, *Presbytis*, *Rhinopithecus*, *Pygathrix*, *Nasalis*, *Simias*), four cercopithecine genera (*Papio*, *Theropithecus*, *Macaca*, *Chlorocebus*), and three hominoid genera (*Homo*, *Pan*, *Pongo*), which were used as outgroup taxa. To complete datasets, we partly implemented sequences from GenBank (Additional file 10). Alignments for individual loci were generated with MAFFT v6 [78] and corrected by eye. In all alignments, poorly aligned positions and indels were removed with Gblocks v0.91b [79] using default settings (Additional file 8). For the mitochondrial dataset, also the D-loop region was excluded (dataset mtDNA1) and a second alignment, generated in Mesquite v2.6 [80], included solely protein-coding genes (dataset mtDNA2). For all datasets, uncorrected pairwise differences were estimated in PAUP (Additional file 7). Nucleotide composition for all and only parsimony-informative positions for the combined nuclear and both mitochondrial alignments was also estimated in PAUP (Additional file 4). To test whether datasets can be combined, we performed partition homogeneity tests in PAUP with 10,000 replications.

Phylogenetic trees were constructed with MP and NJ algorithms as implemented in PAUP as well as with ML and Bayesian algorithms, using the programs GARLI v0.951 [81] and MrBayes v3.1.2 [82,83]. For MP analyses, all characters were treated as unordered and equally weighted throughout. A heuristic search was performed with the maximum number of trees set to 100. For NJ, ML and Bayesian reconstructions, the optimal nucleotide substitution models for each locus and concatenated datasets were chosen using AIC as implemented in MODELTEST v3.7 [84] (Additional file 8). Relative support of internal nodes was assessed by bootstrap analyses with 10,000 (MP, NJ) or 500 replications (ML). In GARLI, only the model specification settings were adjusted according to the respective concatenated dataset, while all other settings were left at their default value. ML majority-rule consensus trees were calculated in PAUP. For Bayesian reconstructions, the datasets were partitioned treating each locus separately and each with its own substitution model. The solely protein-coding alignment of the mitochondrial genome (mtDNA2) was partitioned into codon positions. We used four independent Markov Chain Monte Carlo (MCMC) runs with the default temperature of 0.1. Four repetitions were run for 10,000,000 generations with tree and parameter sampling occurring every 100 generations. The first 25% of samples were discarded as burnin, leaving 75,001 trees per run. PPs for each split and a phylogram with mean branch lengths were calculated from the posterior density of trees.

To evaluate the reliability of obtained relationships among colobines, various alternative tree topologies (Additional file 2) were tested with the Shimodaira-Hasegawa test [85] with full optimization and 1,000 bootstrap replications in PAUP.

### Divergence age estimation

A Bayesian MCMC method, which employs a relaxed molecular clock approach [86], as implemented in BEAST v1.4.8 [87], was used to estimate divergence times. Therefore, a relaxed lognormal model of lineage variation and a Yule prior for branching rates was assumed. Divergence times were calculated for each locus separately and for the combined nuclear dataset. The latter was partitioned treating each locus as distinct unit. The mitochondrial alignment comprising solely protein-coding genes (mtDNA2) was partitioned into codon positions and the substitution model, rate heterogeneity and base frequencies were unlinked across codon positions. Optimal nucleotide substitution models were chosen using AIC in MODELTEST.

As calibrations we used the fossil-based divergence between *Homo *and *Pan*, which has been dated at 6-7 mya [88-90], the separation of *Pongo *from the *Homo*/*Pan *lineage ~14 mya [91], the split between *Theropithecus *and *Papio *~4 mya [92,93], and the divergence of hominoids and cercopithecoids ~24 mya [94-96]. Instead of hardbounded calibration points, we used the published dates as a normal distribution prior for the respective node. For the *Homo *- *Pan *divergence, this translates into a normal distribution with a mean of 6.5 mya and a standard deviation (SD) of 0.5 mya, for the separation of *Pongo *from the *Homo*/*Pan *clade into a mean of 14.0 mya and a SD of 1.0 mya, for the *Theropithecus *- *Papio *split into a mean of 4.0 mya and a SD of 0.5 mya, and for the hominoid - cercopithecoid divergence into a mean of 24 mya and a SD of 2 mya.

Since the estimation of phylogenetic relationships was not the main aim of this analysis, we used an a-priori fixed tree topology as obtained from mobile elements (Figure 1A) for the calculation from nuclear sequence data. Four replicates were run for 10,000,000 generations with tree and parameter sampling occurring every 100 generations. The adequacy of a 10% burnin and convergence of all parameters were assessed by visual inspection of the trace of the parameters across generations using TRACER v1.4.1 [97]. Subsequently, the sampling distributions were combined (25% burnin) using the software LogCombiner v1.4.8 and a consensus chronogram with node height distribution was generated and visualized with TreeAnnotator v1.4.8 and FigTree v1.2.2 [98].

### Inferring hybridization in the presence of incomplete lineage sorting

Statistical support for putative hybridization scenarios was assessed with the method proposed by Kubatko [40], in which statistical model selection techniques (e.g., AIC) are used to compare species trees that may or may not include hybridization scenarios. For our data, we hypothesized two possible hybridization events (for details see Results). The estimated gene trees used as input were those derived from single locus tree reconstructions (Additional file 3) and branch lengths as estimated in BEAST. To estimate evolutionary rates for individual loci, we followed the suggestion of Yang [99] (see also [100]) and computed for each gene the average pairwise sequence divergence of each ingroup (colobine) sequence to the outgroup (non-colobine) taxa. We then assigned to each locus a rate that was calculated by dividing the mean pairwise divergence for that locus by the median of the entire set of pairwise divergences (Additional file 8). To convert gene tree branch lengths to coalescent units, we considered two effective population sizes, 50,000 and 100,000, and used a generation time of 5 years. Since the results were identical in terms of the trees preferred, we show here the results only for effective population size 50,000. For haploid loci (mitochondrial genome, Y chromosomal loci), we additionally divided the rate by 2 (see [100]). We compared a total of nine species trees (four corresponding to no hybridization, four corresponding to single hybridization events, and one that included both hybridization scenarios, Figure 2). The AIC was computed for each tree using the STEM software [100]. Models with AIC values within 2 of one another were regarded as providing similar fit to the data [41].

## Authors' contributions

CR designed the study, collected samples, did laboratory work, analyzed data, and wrote the paper. DZ designed the study, analyzed data, and wrote the paper. LSK, JX, MAB and MB analyzed data and wrote the paper. CS, MY, DM did laboratory work. SDN and LW wrote the paper. FHL, TZ, DPF and TN provided valuable samples and wrote the paper. MO did laboratory work, analyzed data and wrote the paper. All authors read and approved the final manuscript.

## Supplementary Material

Additional file 1 **Additional Table 1**. Presence/absence pattern, location, primers and PCR product sizes of mobile elements Click here for file

Additional file 2 **Additional Table 2**. Alternative tree topology tests Click here for file

Additional file 3 **Additional Figure 1**. Single-locus phylogenetic trees (80% majority rule) Click here for file

Additional file 4 **Additional Figure 2**. Nucleotide composition of both mitochondrial and the combined nuclear datasets Click here for file

Additional file 5 **Additional Figure 3**. Phylograms based on the mitochondrial and combined nuclear datasets Click here for file

Additional file 6 **Additional Table 3**. Divergence ages in mya estimated for each locus separately Click here for file

Additional file 7 **Additional Table 4**. Uncorrected pairwise nucleotide differences for each locus Click here for file

Additional file 8 **Additional Table 5**. Locus-specific information including alignment length, number of variable sites, selected substitution model and estimated evolutionary rates Click here for file

Additional file 9 **Additional Table 6**. Primers and PCR conditions for the amplification of nuclear loci Click here for file

Additional file 10 **Additional Table 7**. GenBank accession numbers Click here for file
