# Supplementary material for: Nuclear versus mitochondrial DNA: evidence for hybridization in colobine monkeys
Source: BMC Evol Biol. 2011 Mar 24;11:77. doi: 10.1186/1471-2148-11-77 (PMC3068967; doi:10.1186/1471-2148-11-77)
Supplement: Additional file 1 — Additional Table 1. Presence/absence pattern, location, primers and PCR product sizes of mobile elements [file 1471-2148-11-77-S1.PDF]

Additional Table 1. Presence/absence pattern, location, primers and PCR product sizes of mobile elements

Name	MM	PC	CG	PB	PV	SE	TO	PM	RA	PN	NL	SC	Forward	Reverse	Filled	Empty	AT	Locus position (Homo, hg18)	Reference
**Colobinae (20)**																			
**DoucL_Yd_14Re**	0	0*	1*	1	1*	1*	1*	1		1*	1*		TTTTAAAATGTATGCTCCTTGTCA	TCACTGAACTCCTAAATCAGCA	468	158	58	chr11:68568822	1
**DoucL_Yd_21**	0	0*	1*	1	1*	1*	1*	1		1*	1*		TGGGAAGTTTGAAGCCTGATA	TGAAGTTCAAAGGCTTAGTTTTATTTT	792	482	58	chr14:52024695	1
**DoucL_Yd_16**	0	0*	1	1	1*	1*	1*	1		1	1*		CCCTCTGAGCTCCTTCTGAAT	TGGGATCAGTCTTTGCTGACT	566	256	58	chr2:59962224	1
**DoucL_Yd_28**	0	0*	1*	1	1*	1*	1*	1		1*	1*		TTGAAAGAACAGGGGAAATCA	TATCTGACAGCCCTTGACCTG	685	375	58	chr20:30348863	1
**Kirk_Yd_2Re**	0	0*	1*	1	1*	1*	1*	1		1*	1*		GCCTCACATTCATCTTCCAAAc	TCTATGGTTTCTGGAGGTGCTT	530	220	58	chr4:7697083	1
**Kirk_Yd_33Re**	0	0*	1*	1	1*	1*	1*	1*		1*	1*		CTCACAATTTCAGTCTTGTGTGA	GGCCTCAGAGAAGAGACTTTCC	464	154	58	chrX:127625240	1
**SLL_Yd_1**	0	0*	1*	1	1*	1*	1*	1		1*	1*		TTTGGGGAACTTACGGTCTTT	GAGAAGCCACTCACCATTTGA	554	244	58	chr15:24453270	1
**SLL_Yd_8**	0	0*	1	1	1	1*	1*	1		1	1*		CCACCCTTTCCTAAATTTCCA	GTCTTGGCTTTCCCTTCTACG	510	200	50	chr16:46418257	1,2
**SLL_PY2_25**	0	0*	1*	1	1*	1*	1*	1		1*	1*		CCTGAGGCTGCTCAGAGAAA	TGCTTATACGAGGCAACCTTTA	684	374	58	chr17:56469575	1,2
**Nasalis_PY2_28**	0	0*	1	1	1	1*	1*	1		1*	1		GTGCTAATCCTTGGCCACAT	GAATCTGTCTGCCAAAGTCACA	487	177	60	chr2:116383321	1
**N21**	0*	0*	1*	1	1*	1*	1*	1*		1*	1*		ACCACTTTTGCTTGGTTCTCAT	TGTCCAGACCAAGAGACATCTG	567	257	58	chr2:242112250	1,2
**N16**	0*	0*	1*	1	1*	1*	1*	1		1*	1*		CAGATTCAAAGAGGTGATGTCG	TAGGTGTCTCAGGATGGTGCTA	540	230	58	chr11:56721243	1,2
**2499**	0		1	1	1*	1	1	1		1	1		CTTCTTTATCTTCTGTATGCCC	GTGGTCCTAGCCTATGCTG	740	410	58	chr1:43201455	this study
**3133**	0		1*	1	1	1*	1*	1*		1	1		TGTCTTCTTCCCTCTGGAAATCTA	GTGGTCCCCAATCTCCAAG	450	150	58	chr20:57502278	this study
**3141**	0		1*	1	1	1*	1*	1*		1*	1*		CCAGTGTTTTGGTGTCAAAATG	GGGGTTTAAGGTTAATGATGACAG	550	250	58	chr12:4069255	this study
**3249**	0		1	1	1	1	1	1		1	1		ATGTCTTGAGAGTTCAGGGC	CTTAAGATACACAATATAACAAGC	600	300	58	chr2:203941433	this study
**3261**	0		1	1	1	1	1	1		1	1		GTAATATAGCTTGGAAAGATGC	TTATTCTGTGACTTGGAAATAGT	600	300	58	chr2:212208212	this study
**3269**	0		1	1	1	1	1	1		1	1		TGTAGCCAGGGAAGCCTCT	TGGGATTTCTAATACTATGCCTTTG	800	500/350	58	chr11:12743945	this study
**3365**	0		1	1	1	1	1	1		1	1		GTCTTCTTCCCTCTGGAAATCT	AGCTGCCTGGATGAGACCT	500	200	58	chr20:57502300	this study
**3369**	0		1	1	1	1	1	1		1	1		TGGTTTCATGTGTCCACTTAGG	CCAAGAATTTATTGAGCATCCA	900	600	58	chr17:35395755	this study
**Piliocolobus + Procolobus (2)**																			
**6169**	0		0	1	1	0	0*	0		0	0		GACAAATCTGATAATCTTATAGG	CAGCAAGAACTCTTTGCATTC	720	400	58	chr15:57476905	this study
**6023**	0	0	0	1	1	0	0	0		0	0		ACAACCAGAACCAAAGAGTAG	GTTCAGATTCAAACTTTAAGTTG	1960	200	58	chrX:78092011	this study
**Piliocolobus/Procolobus + Asian colobines (3)**																			
**2474**	0		0	1	1	1	1	1		1	1		TGGACAAGCTGAAGACATGG	CTGGATCTAGAGCTAGCTAG	500	200	58	chr6:4376475	this study
**3371**	0		0	1	1	1	1	1		1	1		CAGAGTGCTAAATTCATGCTTC	TGGCTGTTCCAAAGTCAGTTAG	600	300	58	chr9:111700546	this study
**3373**	0		0	1	1	1	1	1		1	1		TCGTTTGAAGATTTCAGTTGG	CTCTGTCTTGACAGCAGTAAC	600	300	58	chr1:8704528	this study
**Asian colobines (30)**																			
**DoucL_PY2_3**	0	0*	0*	0	0	1*	1	1		1	1*		TGCGTATTTCCACATTTCTGAC	GGCACAGCAAATGACTACGTTA	560	250	55	chr3:44778763	1
**DoucL_PY2_12RE**	0	0*	0*	0	0	1	1*	1		1	1		CCTTGATTTCATCTATGGGCTTA	TGACAAGGGAAATAGAAAGATTGA	743	433	60	chr3:32047343	1
**DoucL_PY2_21a**	0	0	0	0	0	1	1	1		1	1		CACATTAGAGGGCGCTAGAGTT	TCCTGTTGGTCCTTTGGTAGTT	574	264	55	chr20:41704352	1
**Nasalis_PY2_36**	0*	0*	0*	0	0*	1*	1*	1*		1*	1*		CAGAAATGTGTTTGTGAAGCAG	ACCCTAATGGCAACATTCAGTT	706	396	60	chr3:194023457	1
**DoucL_Yd_2RE**	0	0*	0*	0	0	1	1	1		1	1		TCCCACTTCCTCTTTTTCTCAG	CTTTTATGGTTTTGCGGCTAAT	578	268	60	chr2:158707448	1
**DoucL_Yd_5RE**	0	0*	0	0	0	1	1	1		1	1		ATGTGAAGACCTCTGCCCAGTA	TCCTTTTGTTCAAACTGCTTCTT	440	130	60	chr8:123230272	1,2
**DoucL_Yd_10**	0	0	0	0	0	1*	1*	1		1	1*		TCAAAGAAGCAGCCTTCAAAA	TGCAAAACTCATCTGTGCTGT	610	300	50	chr4:154132124	1
**DoucL_Yd_20**	0	0*	0*	0*	0*	1	1	1	1	1	1		TCAAAACTTGCATTTCTTCACAA	CACTTTCAAAATTGCATGAACA	680	370	58	chr11:24289704	1
**DoucL_Yd_22**	0	0*	0	0	0*	1*	1*	1		1*	1*		TTTCATTTAACCCTCCAGCTATTT	TAACCAAACCTGGCCAGAAC	789	479	58	chr3:38767096	1
**DoucL_Yd_27**	0	0*	0	0	0	1	1	1	1	1*	1*		CAAGGGAGGATTCTAAGTCAGG	CAAATACCTCTTGAGAGCCAGTG	624	314	60	chr8:57979101	1
**PFL_PY2_7**	0	0*	0*	0	0	1	1	1		1	1		ATCCTGCGGTTCACATTTCTAT	GCCAACTTCTTAGAAACAACAGG	560	250	55	chr2:1049801	1
**PFL_PY2_9**	0	0*	0*	0	0	1	1	1		1	1		TTCTAAGCAGGACCTAAAAAGCA	TCATTTCAGAGATTTTGCTGATG	561	251	55	chr6:123355226	1,2
**PFL_Yd_1**	0	0*	0*	0	0	1*	1*	1		1	1*		GTCAGACAAGGTGTGGAACAA	AATGGTTATGTTTGATCTCTTTAACA	466	156	50	chr14:63230793	1
**PFL_Yd_11**	0	0*	0*	0	0	1*	1*	1		1	1*		CAAATGCTAACCACCTTGCAT	GCCCCATCAAAGAATGTATTTC	555	245	48	chr11:75528642	1
**PFL_Yd_15**	0	0*	0*	0	0	1*	1*	1		1*	1*		TTCAGTTTCATCTGGGAGTGG	CATGTGGAGTGACCTGTGTTG	407	97	50	chr15:63007801	1
**SLL_Yd_2**	0*	0*	0	0	0	1	1	1		1*	1*		AACACATCAACACATGCCTCA	CCTTTGGGTTACTCTCCAGGT	645	335	57	chr2:149525794	1
**Sll_Yd_20**	0	0*	0*	0	0	1*	1*	1		1*	1*		TGGTTAAGTAAAGGGGGTGCT	TCATGCTACAACCACAAGCTG	574	264	60	chr1:216592546	1
**Nasalis_PY2_27**	0	0*	0*	0	0	1	1	1		1	1		CAAATGTTCCGTTGAGTCCA	GAGTCTTGGAAGATGCAGTGA	688	378	60	chr6:43076825	1,2
**Nasalis_PY2_32**	0	0*	0*	0	0	1*	1*	1		1*	1*		TTTTGTAAACAGCCAAAGCTCA	TTGTTGAAAATATGGCACAAGC	624	314	55	chr10:50351825	1,2

**3125**	0		0	0	0	1	1	1		1	1		AAGAATCCCAGGGAAGAACACT	TTGCTGGCAAAGTGACTCCT	700	400	58	chr7:107109056	3
**3131**	0		0	0	0	1	1	1		1	1		CCTGCCACTTCTGTCCATCT	AGAACAAACACCAAGACAACAGC	450	150	58	chr3:193991928	3
**3149**	0		0	0	0	1	1	1		1	1		GCTTTGCCACATAAAGAGCTG	GGTTAGGTGCAAATGGGAAAC	420	120	58	chr2:109178381	3
**3247**	0		0	0	0	1	1	1		1	1		TCAATCTTCCAGGGAAAATAAAG	GAATATTAGTTGAAATATTTAGGC	600	300	58	chr15:48692337	3
**3253**	0		0	0	0	1	1	1		1	1		GACCATGGTAAGACAAATGTG	GACTCAGGCTTAATTTTAAGTC	500	200	58	chr4:39427060	3
**3267**	0		0	0	0	1*	1	1		1	1		CACCAAGCACAACTGTGAGG	TCTGCCATAGCCATCAGTCA	600	300	58	chr1:217214400	3
**3377**	0		0*	0	0	1	1	1		1	1		CTCTTGGTTGGGGTGAAGC	GATGGTTGAACAGTGAGACTTGA	500	200	58	chr10:119562900	3
**2482**	0*		0	0	0*	1	1	1		1	1		ACTGAAACTGGTTCATGTGAC	GTTAGGGATCGTTTCCTCAG	490	160	58	chr3:180793781	this study
**2490**	0*		0	0	0*	1	1	1		1	1		CATCTGTGGATCTGAAGCAG	TAAGAAACTACATGGAAAAGCC	540	194	58	chr12:25743239	this study
**3379**	0		0*	0	0	1	1	1		1	1		AGCACCATCAGGCACTCACT	GGGAGATTGGGAAATGGAGT	700	400	58	chr6:104107889	this study
**6580**	0	0*	0*	0*	0*	1*	1*	1*	1*	1*	1*		GTTTGTTAAATGATGAGAAAAGA	CTCTAGAGAGTGGGCAGG	460	160	56	chr12:21646931	this study
**Presbytis + odd-nosed monkeys (2)**																			
**DoucL_PY2_22**	0	0*	0*			0	0	1	1	1	1		TGGTGCTCACTATCAGCAATTT	TGCTTAAAGTCCATCAACATGC	524	214	58	chr12:18654751	2
**3277**	0		0		0	0	0	1	1*	1	1		ACCTTGATCTCAGGGATCCT	GGTCAAAGTCCTACTTAAGGA	410	110	58	chr7:20757072	this study
**odd-nosed monkeys (5)**																			
**DoucL_PY2_13RE**	0	0*	0*			0	0	0	1	1	1		CAATGGGGACAGCTATTTTTCT	CTTTGGGTGTGTAGTTCCCATT	451	141	58	chr13:104854570	1
**DoucL_Yd_36**	0	0*	0*			0	0	0	1	1	1		CTCAAGCTTCTCCCCTCCTTA	AAGGCACAGGATTCTGCTTTT	434	124	58	chr11:20035364	1
**Nasalis_PY2_23**	0	0*	0*			0*	0	0	1	1	1		GGGAAAAGGATGAGTGAATCTG	TCTTCTCATAGGATGCCAGTCA	700	351	58	chr4:86118446	1
**3143**	0		0			0	0	0	1	1	1		AGAAAGTCCCTCCCCAACAC	AAGTTGGCAAAGTGGATTGC	550	250	58	chr1:201638132	3
**6578**	0	0*	0*			0*	0*	0*	1*	1*	1*		CAATAAACTCTGCTTAATTTATC	ATTCCTCAACCTTGGCCAG	430	130	56	chr15:40251990	this study
**Pygathrix + Nasalis/Simias (2)**																			
**3381**	0					0	0	0	0	1	1		GCATGATAAGAGTGGAAATCTGTG	TCAACTGATGCAGAAAATGC	500	200	58	chr18:3757704	this study
**DoucL_Yd_33**	0	0*	0*			0	0	0	0	1	1		CTGGCACCCAACATTGTACTT	TTGCACTTCCATCTTCATTGC	610	300	58	chr13:56959102	1
**Nasalis + Simias (9)**																			
**Nasalis_PY2_24**	0	0*	0*			0*	0*	0	0	0*	1	1*	TTATGGGCCCAATTTAAGTTTT	ATAAAATGGACTTGCCAGATGC	650	340	58	chr7:89850261	1,2
**Nasalis_PY2_25**	0	0*	0*			0*	0*	0	0	0*	1*	1*	TGCTCCCATTTATGAGGATTTT	ATGGGGTTGTCTGTCTGTCTCT	661	351	58	chr12:97767677	1,2
**Nasalis_PY2_33**	0	0*	0*			0*	0*		0	0*	1*	1*	CAGTTAGGTGGCTTAGGGAAAA	TCCTATTGGCATTAAAGCATGA	628	318	58	chr17:48297340	1,2
**3127**	0		0			0	0	0	0*	0	1	1*	TGCATTTATCTTCCCCTTCTTCC	TGGAGCTCCCTCTGTCCTCT	500	200	58	chr11:127217407	this study
**3145**	0*		0*			0*	0*	0*	0*	0*	1*	1*	CTTAGAGCTCAGATTGTGTTTATGACC	TGACAGCAGCACACTGCATA	623	323	58	chr4:152420536	this study
**3153**	0*		0*			0*	0*	0*	0*	0*	1*	1*	GGAACCCAGAAGGATATTAGCAG	CCTATATTAGAGCACCATCTTGTGG	590	287	58	chr7:26371431	this study
**3367**	0		0			0	0	0	0*	0	1	1*	CCAGTGAGACAACATGTGGAA	CCAGAATGTATTTCTATTGATGAG	550	250	58	chr5:18542195	this study
**3375**	0		0			0	0	0	0*	0	1	1*	CCTGTGCATTCTTGGGATAAC	AGCAGAAGGCCTTGATTCAG	700	400	58	chr2:239017222	this study
**6073-6022**	0		0			0	0	0	0	0	1	1*	CTTCGCTCTCAATCTTCCCC	CAGGAAACAGCAGAGTTTCC	717	390	58	chrX:78091960	thit study
**Trachypithecus + Semnopithecus (10)**																			
**Sll_Yd_18**	0	0*	0*			1	1	0		0	0		GCATTCTGGACAGTGGTGATT	GACGAGATACAATGCTTCTGAAA	897	587	60	chr5:50538553	1
**PFL_PY2_14RE**	0	0*	0*			1	1	0		0*	0*		TGCCAAAAACTCAGGTTAAGAGA	ATTTTGGGGGAAAACTGCTATC	563	253	60	chrX:14789579	1,2
**SLL_PY2_23**	0	0*	0*			1	1	0		0	0		ATAGCCTGCACGAAAAGACCTA	CAGGAGTGTTTCTCATTGACCA	513	203	60	chr22:16311986	1
**PFL_PY2_2**	0	0*	0*			1	1	0		0	0		CATGCTCACCTTGTATTCCTTG	TGAGGTACTGCTCTGGTGAGTT	664	354	55	chr11:111606847	1,2
**SLL_PY2_17**	0	0*	0*			1	1	0		0	0		TGATCCATCCCTCTTAGGAGTC	AGATCTCGGTGCCACAAATAGT	694	384	50	chr12:118218700	1,2
**2457**	0		0*			1	1	0		0	0		TGATTAAAGTCAGATGAACACC	GTGTAATGGGATGAAGAACAC	540	240	58	chr15:52962566	3
**2652**	0		0*			1	1	0		0	0		ATACATAGCATTGACTTAACTCT	GATCCTGAGCCCACTATTCT	520	220	58	chr5:25601752	3
**2668**	0		0*			1	1	0		0	0		ACATCAGTGACATCAAATAAGG	GAGGAAAAGATACTTTCTCATG	470	170	58	chr8:95826066	3
**3257**	0		0			1	1	0		0	0		GGATTGAGAGCAATTTTAAAAGGA	GTTCACTCCCAAATCATACTTC	940	640	58	chr15:63693377	3
**3269**	0		0			1	1	0		0	0		TGTAGCCAGGGAAGCCTCT	TGGGATTTCTAATACTATGCCTTTG	800	500/350	58	chr11:12743945	3

Abbreviations: MM, *Macaca mulatta*; PC, *Papio cynocephalus*; CG, *Colobus guereza*; PB, *Piliocolobus badius*; PV, *Procolobus verus*; SE, *Semnopithecus entellus*; TO, *Trachypithecus obscurus*; PM, *Presbytis melalophos*; RA, *Rhinopithecus avunculus*; PN, *Pygathrix nemaeus*; NL, *Nasalis larvatus*; SC, *Simias concolor*; AT, Annealing temperature; 1, presence of integration; 0, absence of integration; 1/0, sequenced; 1*/0*, not sequenced; Reference 1, Xing et al. 2005; Reference 2, Herke et al. 2007; Reference 3, Osterholz et al. 2008
