# Supplementary figures and images for: Nuclear versus mitochondrial DNA: evidence for hybridization in colobine monkeys
Source: BMC Evol Biol. 2011 Mar 24;11:77. doi: 10.1186/1471-2148-11-77 (PMC3068967; doi:10.1186/1471-2148-11-77)

**Additional Figure 1.** Single-locus phylogenetic trees (80% majority rule)

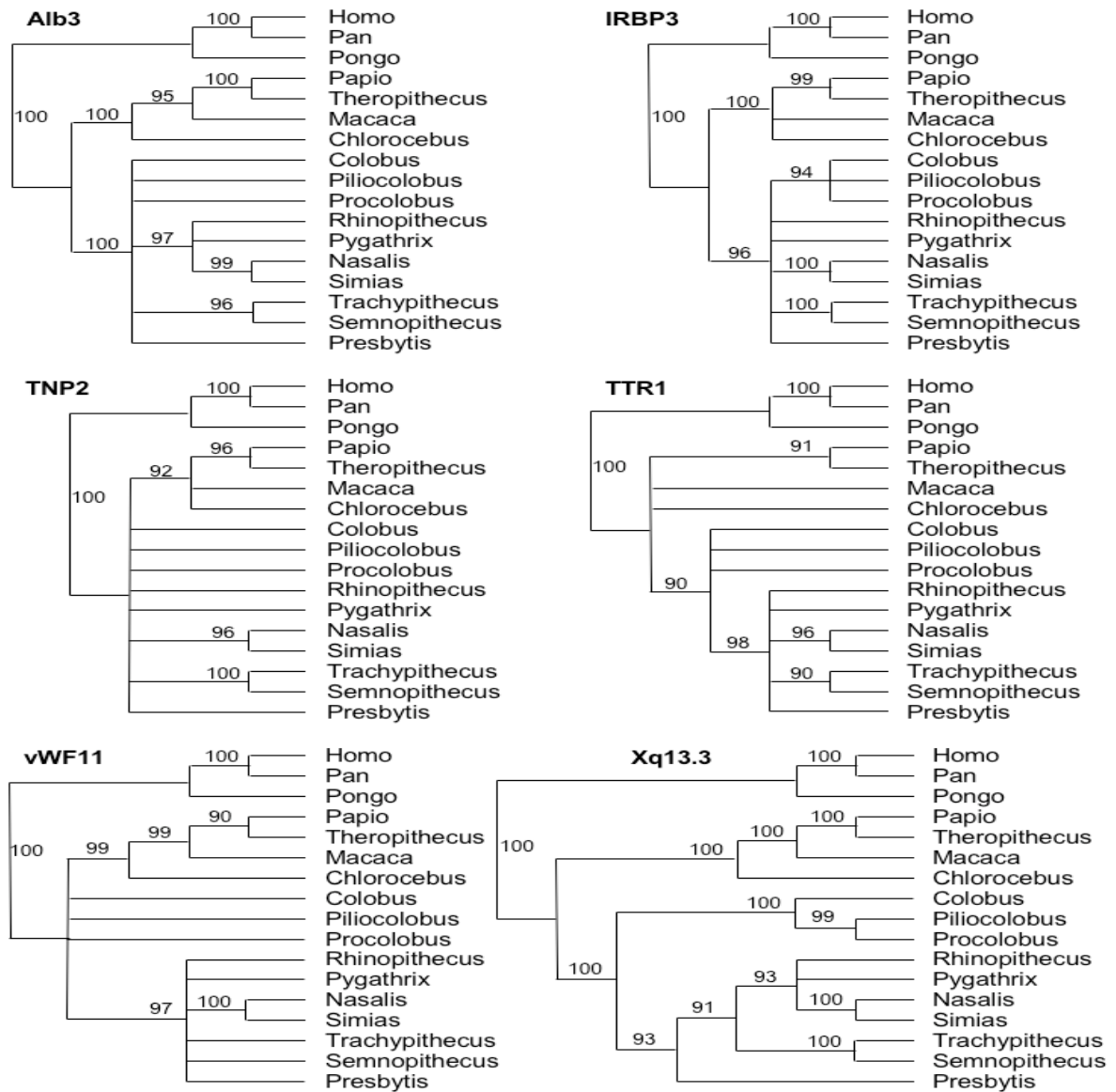

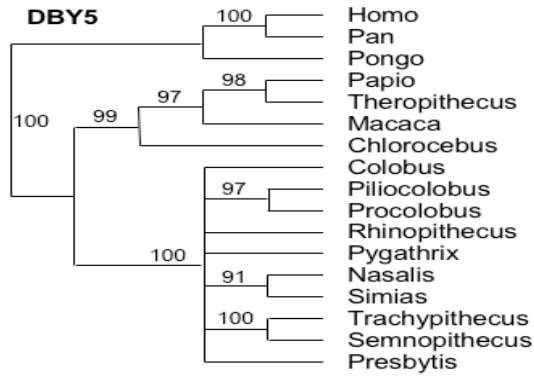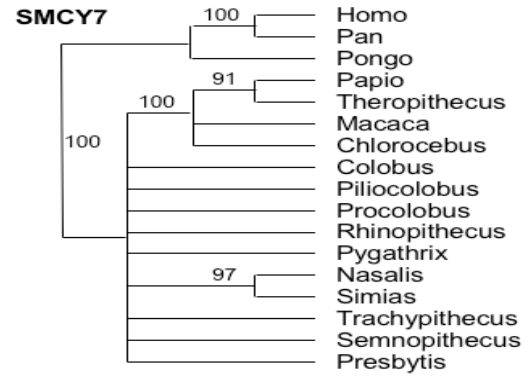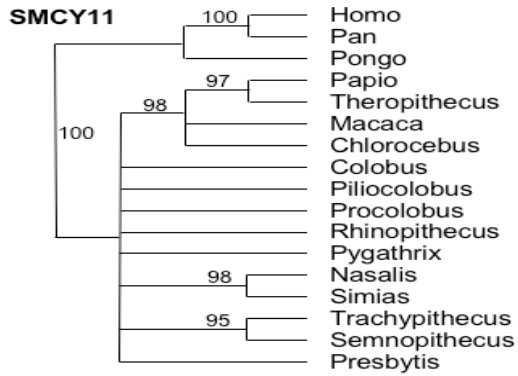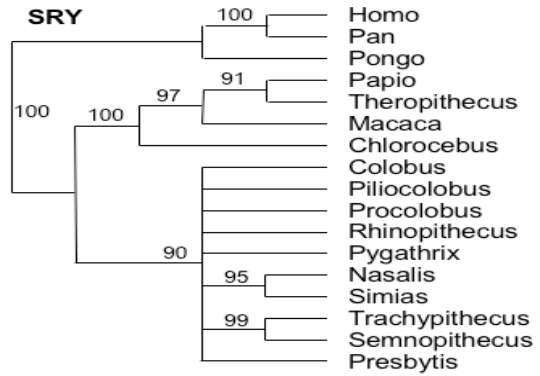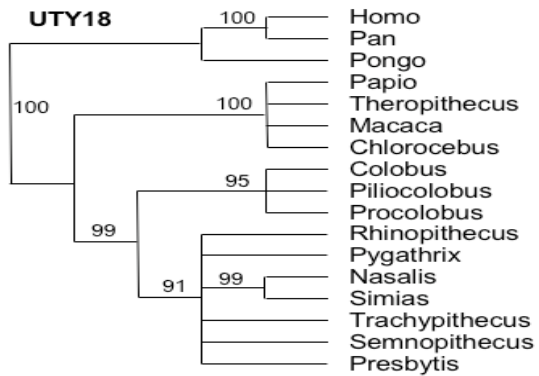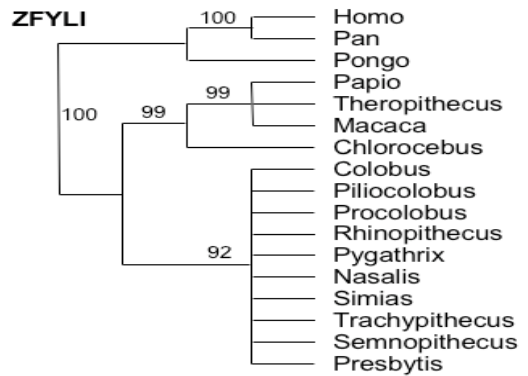

Supplement: Additional file 3 — Additional Figure 1. Single-locus phylogenetic trees (80% majority rule) [file 1471-2148-11-77-S3.PDF]
