# Supplementary material for: Nuclear versus mitochondrial DNA: evidence for hybridization in colobine monkeys
Source: BMC Evol Biol. 2011 Mar 24;11:77. doi: 10.1186/1471-2148-11-77 (PMC3068967; doi:10.1186/1471-2148-11-77)

Additional Figure 2. Nucleotide composition of both mitochondrial and the combined nuclear datasets

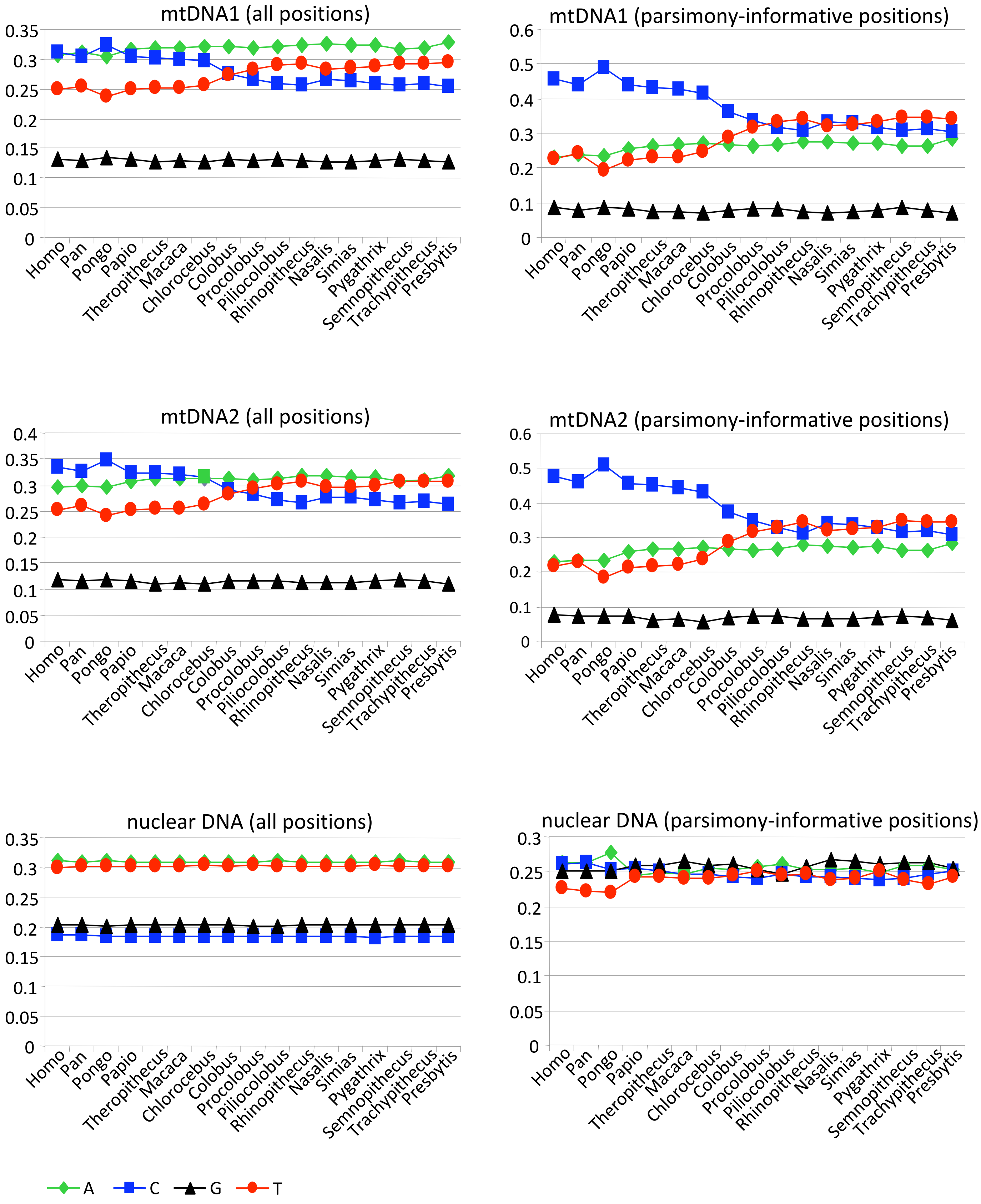

Supplement: Additional file 4 — Additional Figure 2. Nucleotide composition of both mitochondrial and the combined nuclear datasets [file 1471-2148-11-77-S4.PDF]
