# Supplementary material for: Nuclear versus mitochondrial DNA: evidence for hybridization in colobine monkeys
Source: BMC Evol Biol. 2011 Mar 24;11:77. doi: 10.1186/1471-2148-11-77 (PMC3068967; doi:10.1186/1471-2148-11-77)

Additional Figure 3. Phylogram based on the mitochondrial dataset (mtDNA2):

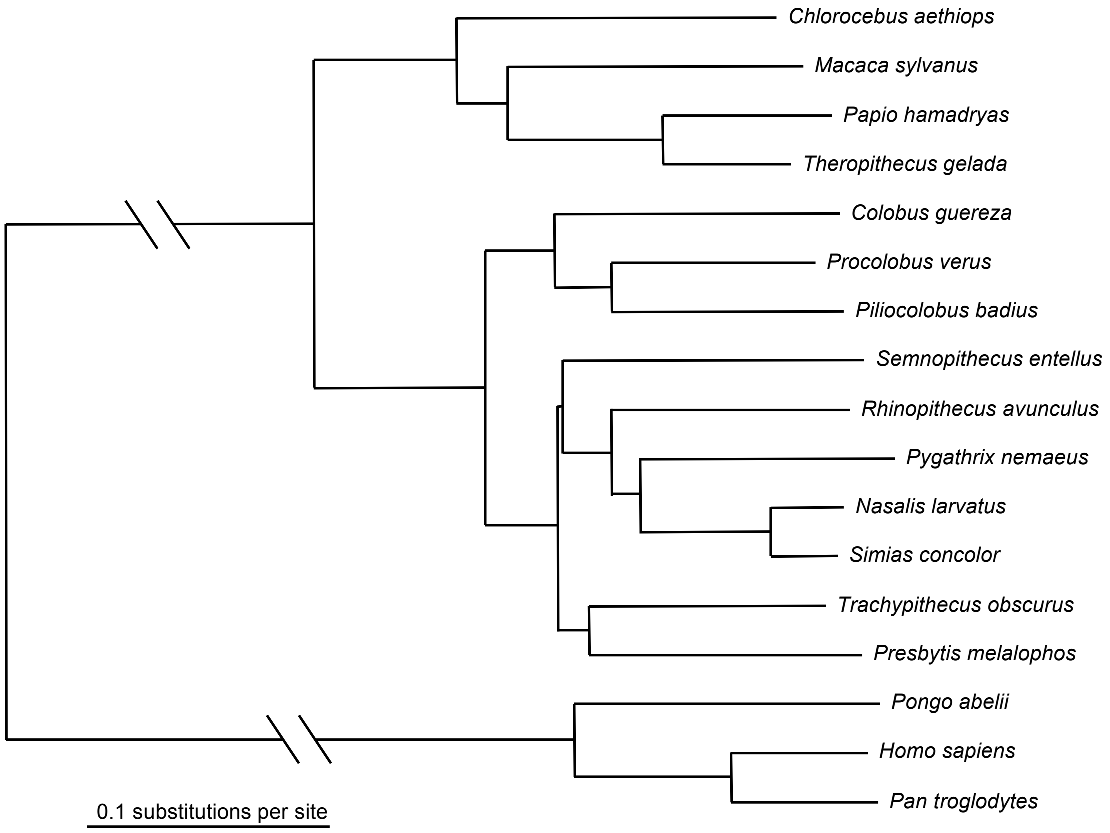

Phylogram based on the combined nuclear dataset:

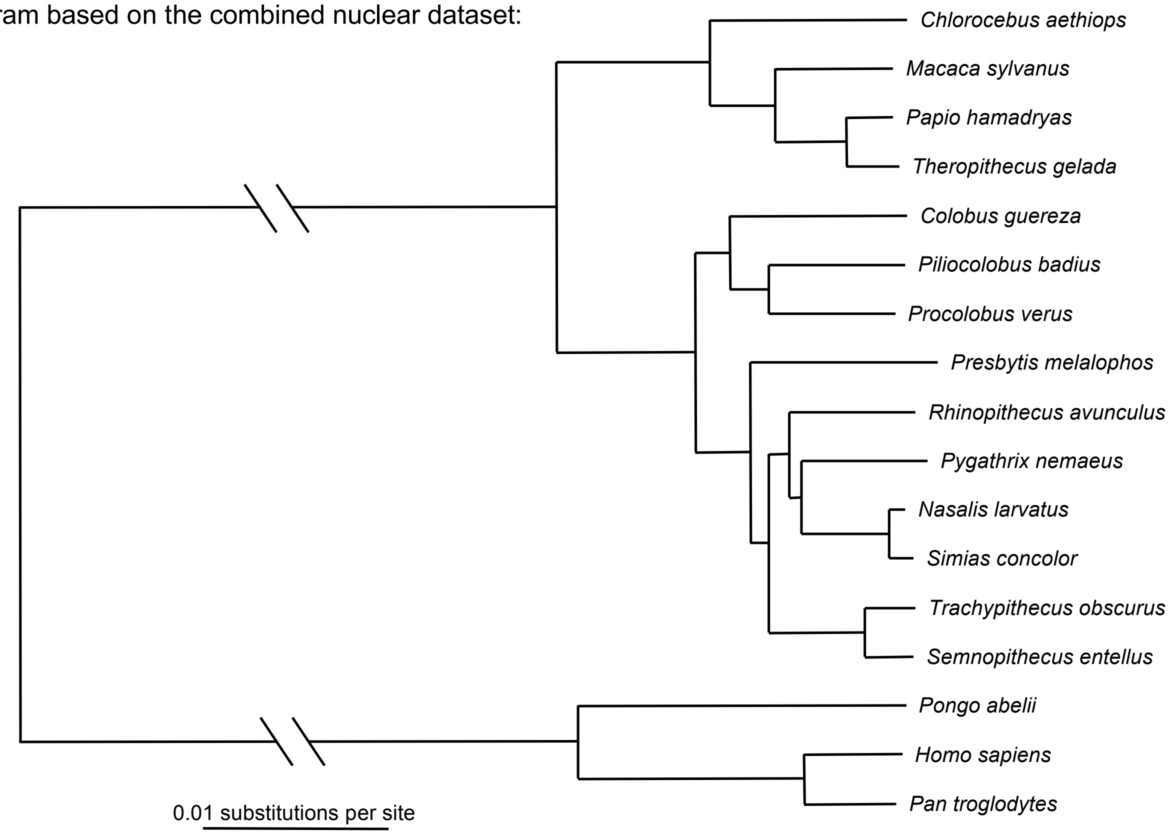

Supplement: Additional file 5 — Additional Figure 3. Phylograms based on the mitochondrial and combined nuclear datasets [file 1471-2148-11-77-S5.PDF]
