# Supplementary material for: Nuclear versus mitochondrial DNA: evidence for hybridization in colobine monkeys
Source: BMC Evol Biol. 2011 Mar 24;11:77. doi: 10.1186/1471-2148-11-77 (PMC3068967; doi:10.1186/1471-2148-11-77)
Supplement: Additional file 7 — Additional Table 4. Uncorrected pairwise nucleotide differences for each locus [file 1471-2148-11-77-S7.PDF]

Additional Table 4. Uncorrected pairwise nucleotide differences for each locus

**Alb3**	CGUE	PBAD	PVER	RAVU	PNEM	NLAR	SCON	PMEL	TOBS
PBAD	0.0156								
PVER	0.0147	0.0113							
RAVU	0.0233	0.0182	0.0190						
PNEM	0.0190	0.0138	0.0147	0.0112					
NLAR	0.0216	0.0165	0.0173	0.0138	0.0061				
SCON	0.0216	0.0165	0.0173	0.0138	0.0061	0.0000			
PMEL	0.0242	0.0208	0.0182	0.0233	0.0190	0.0216	0.0216		
TOBS	0.0190	0.0138	0.0147	0.0182	0.0138	0.0147	0.0147	0.0190	
SENT	0.0164	0.0130	0.0121	0.0156	0.0112	0.0138	0.0138	0.0164	0.0043

**IRBP3**	CGUE	PBAD	PVER	RAVU	PNEM	NLAR	SCON	PMEL	TOBS
PBAD	0.0137								
PVER	0.0189	0.0150							
RAVU	0.0207	0.0182	0.0214						
PNEM	0.0207	0.0182	0.0208	0.0117					
NLAR	0.0214	0.0202	0.0234	0.0110	0.0149				
SCON	0.0214	0.0202	0.0234	0.0110	0.0149	0.0065			
PMEL	0.0221	0.0209	0.0241	0.0188	0.0163	0.0208	0.0221		
TOBS	0.0233	0.0195	0.0240	0.0156	0.0195	0.0169	0.0169	0.0221	
SENT	0.0246	0.0208	0.0253	0.0182	0.0221	0.0195	0.0195	0.0234	0.0078

**TNP2**	CGUE	PBAD	PVER	RAVU	PNEM	NLAR	SCON	PMEL	TOBS
PBAD	0.0202								
PVER	0.0233	0.0124							
RAVU	0.0279	0.0202	0.0233						
PNEM	0.0264	0.0172	0.0202	0.0109					
NLAR	0.0264	0.0186	0.0217	0.0109	0.0093				
SCON	0.0279	0.0202	0.0234	0.0124	0.0109	0.0016			
PMEL	0.0326	0.0249	0.0279	0.0202	0.0186	0.0186	0.0202		
TOBS	0.0326	0.0249	0.0279	0.0202	0.0186	0.0186	0.0202	0.0248	
SENT	0.0326	0.0249	0.0279	0.0202	0.0186	0.0186	0.0202	0.0248	0.0031

**TTR1**	CGUE	PBAD	PVER	RAVU	PNEM	NLAR	SCON	PMEL	TOBS
PBAD	0.0214								
PVER	0.0248	0.0169							
RAVU	0.0281	0.0214	0.0293						
PNEM	0.0293	0.0225	0.0304	0.0146					
NLAR	0.0259	0.0214	0.0293	0.0113	0.0146				
SCON	0.0259	0.0214	0.0293	0.0113	0.0146	0.0000			
PMEL	0.0260	0.0192	0.0271	0.0090	0.0101	0.0090	0.0090		
TOBS	0.0281	0.0214	0.0293	0.0113	0.0146	0.0113	0.0113	0.0090	
SENT	0.0259	0.0192	0.0271	0.0090	0.0124	0.0090	0.0090	0.0068	0.0045

**vWF11**	CGUE	PBAD	PVER	RAVU	PNEM	NLAR	SCON	PMEL	TOBS
PBAD	0.0332								
PVER	0.0277	0.0332							
RAVU	0.0332	0.0354	0.0376						
PNEM	0.0322	0.0366	0.0388	0.0211					
NLAR	0.0366	0.0367	0.0366	0.0166	0.0134				
SCON	0.0376	0.0376	0.0376	0.0177	0.0144	0.0011			
PMEL	0.0300	0.0311	0.0299	0.0177	0.0167	0.0167	0.0155		
TOBS	0.0277	0.0265	0.0298	0.0144	0.0144	0.0133	0.0144	0.0100	

SENT	0.0321	0.0320	0.0320	0.0188	0.0166	0.0155	0.0166	0.0122	0.0111

**Xq13.3**	CGUE	PBAD	PVER	RAVU	PNEM	NLAR	SCON	PMEL	TOBS
PBAD	0.0157								
PVER	0.0166	0.0142							
RAVU	0.0180	0.0185	0.0199						
PNEM	0.0192	0.0202	0.0206	0.0126					
NLAR	0.0161	0.0171	0.0185	0.0100	0.0111				
SCON	0.0164	0.0173	0.0187	0.0102	0.0114	0.0017			
PMEL	0.0187	0.0197	0.0202	0.0149	0.0164	0.0133	0.0135		
TOBS	0.0195	0.0204	0.0218	0.0142	0.0149	0.0123	0.0126	0.0154	
SENT	0.0185	0.0195	0.0209	0.0133	0.0140	0.0114	0.0116	0.0145	0.0033

**DBY5**	CGUE	PBAD	PVER	RAVU	PNEM	NLAR	SCON	PMEL	TOBS
PBAD	0.0363								
PVER	0.0287	0.0076							
RAVU	0.0272	0.0242	0.0166						
PNEM	0.0257	0.0227	0.0181	0.0166					
NLAR	0.0302	0.0227	0.0151	0.0181	0.0196				
SCON	0.0287	0.0181	0.0106	0.0166	0.0181	0.0045			
PMEL	0.0317	0.0302	0.0242	0.0227	0.0212	0.0257	0.0242		
TOBS	0.0317	0.0257	0.0212	0.0227	0.0242	0.0257	0.0212	0.0272	
SENT	0.0332	0.0302	0.0227	0.0242	0.0257	0.0272	0.0227	0.0287	0.0045

**SMCY7**	CGUE	PBAD	PVER	RAVU	PNEM	NLAR	SCON	PMEL	TOBS
PBAD	0.0162								
PVER	0.0139	0.0023							
RAVU	0.0231	0.0208	0.0185						
PNEM	0.0139	0.0116	0.0092	0.0139					
NLAR	0.0208	0.0139	0.0116	0.0208	0.0116				
SCON	0.0231	0.0162	0.0139	0.0231	0.0139	0.0023			
PMEL	0.0162	0.0139	0.0116	0.0185	0.0116	0.0185	0.0208		
TOBS	0.0162	0.0139	0.0116	0.0162	0.0069	0.0139	0.0162	0.0139	
SENT	0.0162	0.0139	0.0116	0.0162	0.0069	0.0139	0.0162	0.0139	0.0046

**SMCY11**	CGUE	PBAD	PVER	RAVU	PNEM	NLAR	SCON	PMEL	TOBS
PBAD	0.0154								
PVER	0.0055	0.0166							
RAVU	0.0122	0.0351	0.0185						
PNEM	0.0147	0.0314	0.0148	0.0148					
NLAR	0.0169	0.0333	0.0166	0.0166	0.0129				
SCON	0.0193	0.0370	0.0203	0.0203	0.0166	0.0037			
PMEL	0.0227	0.0447	0.0296	0.0296	0.0296	0.0314	0.0351		
TOBS	0.0197	0.0388	0.0222	0.0222	0.0185	0.0203	0.0240	0.0333	
SENT	0.0197	0.0351	0.0185	0.0185	0.0148	0.0166	0.0203	0.0333	0.0074

**SRY**	CGUE	PBAD	PVER	RAVU	PNEM	NLAR	SCON	PMEL	TOBS
PBAD	0.0143								
PVER	0.0078	0.0065							
RAVU	0.0207	0.0220	0.0155						
PNEM	0.0168	0.0181	0.0117	0.0143					
NLAR	0.0155	0.0168	0.0104	0.0130	0.0091				
SCON	0.0168	0.0181	0.0117	0.0143	0.0104	0.0013			
PMEL	0.0194	0.0207	0.0143	0.0220	0.0181	0.0168	0.0181		

TOBS	0.0207	0.0220	0.0155	0.0181	0.0143	0.0130	0.0143	0.0220	
SENT	0.0233	0.0220	0.0181	0.0207	0.0168	0.0155	0.0168	0.0246	0.0078

**UTY18**	CGUE	PBAD	PVER	RAVU	PNEM	NLAR	SCON	PMLE	TOBS
PBAD	0.0121								
PVER	0.0073	0.0048							
RAVU	0.0145	0.0145	0.0097						
PNEM	0.0205	0.0205	0.0157	0.0109					
NLAR	0.0181	0.0181	0.0133	0.0085	0.0133				
SCON	0.0181	0.0181	0.0133	0.0085	0.0133	0.0000			
PMEL	0.0230	0.0230	0.0181	0.0133	0.0205	0.0169	0.0169		
TOBS	0.0169	0.0169	0.0121	0.0073	0.0121	0.0085	0.0085	0.0157	
SENT	0.0181	0.0181	0.0133	0.0060	0.0133	0.0097	0.0097	0.0133	0.0036

**ZFYLI**	CGUE	PBAD	PVER	RAVU	PNEM	NLAR	SCON	PMEL	TOBS
PBAD	0.0118								
PVER	0.0132	0.0118							
RAVU	0.0191	0.0206	0.0206						
PNEM	0.0221	0.0235	0.0235	0.0147					
NLAR	0.0191	0.0206	0.0206	0.0147	0.0088				
SCON	0.0177	0.0191	0.0191	0.0132	0.0103	0.0015			
PMEL	0.0206	0.0221	0.0221	0.0191	0.0221	0.0221	0.0206		
TOBS	0.0162	0.0177	0.0177	0.0088	0.0118	0.0117	0.0103	0.0162	
SENT	0.0162	0.0177	0.0177	0.0088	0.0118	0.0118	0.0103	0.0162	0.0029

**mtDNA1**	CGUE	PBAD	PVER	RAVU	PNEM	NLAR	SCON	PMEL	TOBS
PBAD	0.1361								
PVER	0.1318	0.1162							
RAVU	0.1476	0.1449	0.1402						
PNEM	0.1497	0.1497	0.1457	0.1210					
NLAR	0.1455	0.1465	0.1381	0.1174	0.1223				
SCON	0.1455	0.1418	0.1400	0.1164	0.1211	0.0458			
PMEL	0.1540	0.1501	0.1477	0.1329	0.1389	0.1337	0.1322		
TOBS	0.1464	0.1461	0.1420	0.1302	0.1367	0.1307	0.1323	0.1292	
SENT	0.1548	0.1505	0.1495	0.1350	0.1431	0.1347	0.1355	0.1405	0.1353

**mtDNA2**	CGUE	PBAD	PVER	RAVU	PNEM	NLAR	SCON	PMEL	TOBS
PBAD	0.1565								
PVER	0.1523	0.1363							
RAVU	0.1673	0.1659	0.1593						
PNEM	0.1689	0.1712	0.1664	0.1399					
NLAR	0.1660	0.1682	0.1576	0.1352	0.1429				
SCON	0.1656	0.1630	0.1601	0.1354	0.1415	0.0529			
PMEL	0.1758	0.1726	0.1694	0.1521	0.1592	0.1541	0.1524		
TOBS	0.1676	0.1684	0.1632	0.1494	0.1568	0.1480	0.1509	0.1486	
SENT	0.1760	0.1713	0.1712	0.1532	0.1648	0.1539	0.1560	0.1612	0.1537
