# Supplementary material for: Nuclear versus mitochondrial DNA: evidence for hybridization in colobine monkeys
Source: BMC Evol Biol. 2011 Mar 24;11:77. doi: 10.1186/1471-2148-11-77 (PMC3068967; doi:10.1186/1471-2148-11-77)
Supplement: Additional file 8 — Additional Table 5. Locus-specific information including alignment length, number of variable sites, selected substitution model and estimated evolutionary rates [file 1471-2148-11-77-S8.PDF]

Additional Table 5. Locus-specific information including alignment length, number of variable sites, selected substitution model and estimated evolutionary rates

Locus	Alignment with / without indels	Variable sites / parsimony-informative sites	Substitution model (AIC)	Estimated evolutionary rates
ALB3	1204 / 1157	182 / 93	TVM + I	0.902
IRBP3	1615 / 1543	263 / 114	K81uf + G	0.848
TNP2	894 / 645	120 / 56	GTR	1.000
TTR1	906 / 889	151 / 73	TVM + G	0.953
vWF11	938 / 905	181 / 98	HKY + G	1.156
**autosomal loci combined**	**5557 / 5139**	**897 / 434**	**TVM + G**	
**Xq13.3**	**6634 / 4218**	**621 / 327**	**TVM + G**	**0.906**
DBY5	1048 / 662	144 / 83	GTR + I	1.403
SMCY7	463 / 433	89 / 56	TVM	1.500
SMCY11	606 / 541	108 / 51	TVM	1.080
SRY	786 / 772	123 / 65	TVM	0.948
UTY18	881 / 828	131 / 73	HKY + G	1.082
ZFYLI	714 / 680	106 / 54	TVM	0.921
**Y chromosomal loci combined**	**4498 / 3916**	**701 / 382**	**TVM + G**	
**nuclear loci combined**	**16689 / 13273**	**2154 / 1143**	**TVM + G**	
**mitochondrial genome**	**16860 / 15074*** **16860 / 11316****	**6998 / 5457*** **5745 / 4592****	**GTR + I + G** **GTR + I + G**	**3.511**
**all data combined**	**33549 / 28347*** **33549 / 24589****	**9152 / 6600*** **7899 / 5735****	**-** **-**	

* mitochondrial alignment excluding indels, poorly aligned positions and D-loop

** mitochondrial alignment with only protein-coding genes
