# Supplementary material for: Nuclear versus mitochondrial DNA: evidence for hybridization in colobine monkeys
Source: BMC Evol Biol. 2011 Mar 24;11:77. doi: 10.1186/1471-2148-11-77 (PMC3068967; doi:10.1186/1471-2148-11-77)
Supplement: Additional file 9 — Additional Table 6. Primers and PCR conditions for the amplification of nuclear loci [file 1471-2148-11-77-S9.PDF]

Additional Table 6. Primers and PCR conditions for the amplification of nuclear loci

Locus	Ref.	Forward primer	Reverse Primer	AT
ALB3	-	GCATTCAAAGTCAACCATG	ACGAAGAGTTGCAACTGTGC	56°C
IRBP3	-	CTCTGGACACACGCCCAG	CACACTGCTGGTCAGAATGA	58°C
TNP2	-	GCAGGTGTACAAAACCAAG	GTCTCATTAGTTGGATTTCC	54°C
TTR1	-	GGCCCTACGGTGAGTGTT	ACTTTGACCATCAGAGGACA	56°C
vWF11	[73]	see ref.	see ref.	see ref.
Xq13.3	[10,75]	see ref.	see ref.	see ref.
DBY5	[74]	see ref.	see ref.	see ref.
SMCY7	[74]	see ref.	see ref.	see ref.
SMCY11	[74]	see ref.	see ref.	see ref.
UTY18	[74]	see ref.	see ref.	see ref.
SRY	[72]	see ref.	see ref.	see ref.
ZFYLI	-	CCTGATTCCAGGCAGTACC	ATCAGGGCCAATAATTATTGCT	58°C
