# Supplementary material for: Nuclear versus mitochondrial DNA: evidence for hybridization in colobine monkeys
Source: BMC Evol Biol. 2011 Mar 24;11:77. doi: 10.1186/1471-2148-11-77 (PMC3068967; doi:10.1186/1471-2148-11-77)
Supplement: Additional file 10 — Additional Table 7. GenBank accession numbers [file 1471-2148-11-77-S10.PDF]

Additional Table 7. GenBank accession numbers*

Species	ALB3	IRBP3	TNP2	TTR1	vWF11	DBY5	SMCY7	SMCY11	SRY	UTY18	ZFYLI	Xq13.3	mtDNA
Homo sapiens	*EF649953*	*J05253*	*L03378*	*M11844*	*AC006576*	*AC004474*	*AF273841*	*AF273841*	*X53772*	*AF265575*	*U24118*	*AJ241091*	*X93334*
Pan troglodytes	JF293097	JF293129	JF293193	JF293209	JF293241	JF293113	JF293145	JF293161	JF293177	JF293225	JF293262	*AJ270088*	*D38113*
Pongo abelii	JF293098	JF293130	JF293194	JF293210	JF293242	JF293114	JF293146	JF293162	JF293178	JF293226	JF293263	JF293257	*X97707*
Papio hamadryas	JF293100	JF293132	JF293197	JF293213	JF293243	JF293117	JF293149	JF293164	JF293180	JF293228	JF293265	*AY899234*	*Y18001*
Theropithecus gelada	JF293101	JF293133	JF293196	JF293212	JF293244	JF293116	JF293148	JF293165	JF293181	JF293229	JF293266	*AY899236*	*FJ785426*
Macaca sylvanus	JF293099	JF293131	JF293195	JF293211	JF293245	JF293115	JF293150	JF293163	JF293179	JF293227	JF293264	JF293258	*AJ309865*
Chlorocebus aethiops	JF293102	JF293134	JF293198	JF293214	JF293246	JF293118	JF293147	JF293166	JF293182	JF293230	JF293267	*AY899216*	*AY863426*
Colobus guereza	JF293103	JF293135	JF293199	JF293215	JF293247	JF293119	JF293151	JF293167	JF293183	JF293231	JF293268	*AY899240*	*AY863427*
Piliocolobus badius	JF293104	JF293136	JF293200	JF293216	JF293248	JF293120	JF293152	JF293168	JF293184	JF293232	JF293269	*EU342361*	*DQ355301*
Procolobus verus	JF293105	JF293137	JF293201	JF293217	JF293249	JF293121	JF293153	JF293169	JF293185	JF293233	JF293270	JF293259	JF293092
Trachypithecus obscurus	JF293111	JF293144	JF293207	JF293223	JF293255	JF293127	JF293159	JF293175	JF293191	JF293239	JF293276	*EU342365*	*AY863425*
Semnopithecus entellus	JF293112	JF293143	JF293208	JF293224	JF293256	JF293128	JF293160	JF293176	JF293192	JF293240	JF293277	*EU342364*	*DQ355297*
Presbytis melalophos	JF293106	JF293138	JF293202	JF293218	JF293250	JF293122	JF293154	JF293170	JF293186	JF293234	JF293271	JF293260	*DQ355299*
Nasalis larvatus	JF293108	JF293140	JF293204	JF293220	JF293252	JF293124	JF293156	JF293172	JF293188	JF293236	JF293273	*EU342359*	JF293094
Simias concolor	JF293109	JF293141	JF293205	JF293221	JF293253	JF293125	JF293157	JF293173	JF293189	JF293237	JF293274	JF293261	JF293095
Pygathrix nemaeus	JF293107	JF293139	JF293203	JF293219	JF293251	JF293123	JF293155	JF293171	JF293187	JF293235	JF293272	*EU342362*	JF293096
Rhinopithecus avunculus	JF293110	JF293142	JF293206	JF293222	JF293254	JF293126	JF293158	JF293174	JF293190	JF293238	JF293275	*EU342363*	JF293093

*sequences in italic are taken from GenBank
